# Fine-Mapping the Wheat *Snn1* Locus Conferring Sensitivity to the *Parastagonospora nodorum* Necrotrophic Effector SnTox1 Using an Eight Founder Multiparent Advanced Generation Inter-Cross Population

**DOI:** 10.1534/g3.115.021584

**Published:** 2015-09-24

**Authors:** James Cockram, Alice Scuderi, Toby Barber, Eiko Furuki, Keith A. Gardner, Nick Gosman, Radoslaw Kowalczyk, Huyen P. Phan, Gemma A. Rose, Kar-Chun Tan, Richard P. Oliver, Ian J. Mackay

**Affiliations:** * John Bingham Laboratory, National Institute of Agricultural Botany (NIAB), Huntington Road, Cambridge, CB3 0LE, United Kingdom; † Department of Drug Science and Products for Health, University of Messina, Sicily, 98122, Italy; ‡ Centre for Crop Disease Management, Curtin University, WA6845, Australia; § Faculty of Life Sciences, University of Manchester, M13 9PL, United Kingdom

**Keywords:** fungal protein effectors, diagnostic genetic markers, plant disease resistance breeding, multiparent genetic mapping populations, high-density crop genotyping, MPP, Multiparent Advanced Generation Inter-Cross (MAGIC), multiparental populations

## Abstract

The necrotrophic fungus *Parastagonospora nodorum* is an important pathogen of one of the world’s most economically important cereal crops, wheat (*Triticum aestivum* L.). *P. nodorum* produces necrotrophic protein effectors that mediate host cell death, providing nutrients for continuation of the infection process. The recent discovery of pathogen effectors has revolutionized disease resistance breeding for necrotrophic diseases in crop species, allowing often complex genetic resistance mechanisms to be broken down into constituent parts. To date, three effectors have been identified in *P. nodorum*. Here we use the effector, SnTox1, to screen 642 progeny from an eight-parent multiparent advanced generation inter-cross (*i.e.*, MAGIC) population, genotyped with a 90,000-feature single-nucleotide polymorphism array. The MAGIC founders showed a range of sensitivity to SnTox1, with transgressive segregation evident in the progeny. SnTox1 sensitivity showed high heritability, with quantitative trait locus analyses fine-mapping the *Snn1* locus to the short arm of chromosome 1B. In addition, a previously undescribed SnTox1 sensitivity locus was identified on the long arm of chromosome 5A, termed here *QSnn.niab-5A.1*. The peak single-nucleotide polymorphism for the *Snn1* locus was converted to the KASP genotyping platform, providing breeders and researchers a simple and cheap diagnostic marker for allelic state at *Snn1*.

The necrotrophic wheat pathogen *Parastagonospora* (synonym: *Septoria*, *Stagonospora*, *Phaeosphaeria*) *nodorum* causes the disease Septoria nodorum blotch (SNB), responsible for significant wheat (*Triticum aestivum* L.) yield losses in Europe, Australasia, United States, North America, and North Africa (Friesen *et al.* 2005; Oliver *et al.* 2012). Necrotrophic pathogens derive their energy from dead host plant cells or tissues, in contrast to biotrophs, which obtain their energy from living cells. Recent work indicates that necrotrophic pathogens produce numerous proteinaceous and metabolite molecules, previously known as host-specific (or selective) toxins, now known as effectors, that facilitate pathogen entry by eliciting a necrosis response in the host (Oliver and Solomon 2010). Where the host plant is sensitive to the effector, the resulting cell death provides a rich nutrient source, which promotes necrotrophic infection (Vincent *et al.* 2012). This process is known as effector-triggered susceptibility. In *P. nodorum*, an inverse gene-for-gene interaction operates in which mutations in corresponding effector sensitively loci results in insensitivity and host resistance (Friesen *et al.* 2007). The identification and characterization of effectors in *P. nodorum* (Friesen *et al.* 2007; Oliver and Solomon 2010) and other pathogen species represents a paradigm shift within the field of phytopathology, providing tools with which to break down quantitative host sensitivity phenotypes into constitutive components.

The first necrotrophic protein effector to be isolated was PtrToxA from the wheat tan spot pathogen, *Pyrenophora tritici-repentis* (Ballance *et al.* 1989; Tómas *et al.* 1990), which causes necrosis in wheat lines carrying susceptible alleles at the *Tsn1* locus (Thomas *et al.* 1990; Faris *et al.* 1996). Sequencing of the *P. nodorum* genome (Hane *et al.* 2007) identified a predicted protein almost identical in sequence to PtrToxA. This gene, termed *SnToxA*, encodes a 13-kDa protein containing two cysteine residues and an RGD-containing vitronectic-like motif (Ballance *et al.* 1996; Ciuffetti *et al.* 1997) and is thought to have been transferred from *P. nodorum* to *P. tritici-repentis* by lateral gene transfer (Friesen *et al.* 2006; Stukenbrock and McDonald 2007). ToxA from both species cause necrosis only in wheat lines carrying dominant sensitivity alleles at the *Tsn1* locus (Friesen *et al.* 2006; Faris *et al.* 2010). *Tsn1* has been cloned recently and encodes a predicted protein encoding domains commonly found in plant disease resistance genes: a serine/threonine protein kinase domain, a nucleotide-binding site (NBS), and leucine-rich repeat (LRR) domains (Faris *et al.* 2010). Although a small number of varieties have been identified with point mutations in *Tsn1* that result in ToxA insensitivity (Faris *et al.* 2010), the vast majority of insensitive wheat varieties screened to date carry a deletion of *Tsn1*, allowing the development of a diagnostic molecular marker for allelic state at the locus.

After the identification of SnToxA, additional *P. nodorum* effectors have been isolated. The first of these was SnTox3, which encodes a 230 amino acid cysteine rich pro-protein, containing a predicted signal sequence and prosequence of 20 and ∼30 amino acids, respectively. SnTox3 causes necrosis in wheat lines carrying sensitive alleles at the *Snn3* locus on chromosome 5BS (Liu *et al.* 2012). The SnTox3−*Snn3* interaction has been shown to account for a significant proportion of disease phenotype in adult plants segregating for sensitivity alleles at *Snn3*, *Tsn1*, and *Snn2*, although only using *P. nodorum* isolates lacking *SnToxA* (Friesen *et al.* 2008), indicating the SnToxA-*Tsn1* interaction is epistatic to the SnTox3−*Snn3* interaction. SnTox1 is the most recent *P. nodorum* effector to be identified. It encodes a protein of 10.33 kDa and causes necrosis on wheat lines carrying dominant sensitive alleles at the *Snn1* locus (Liu *et al.* 2012). *SnTox1* was identified by screening a list of candidate genes identified in the *P. nodorum* genome has possessing characteristics common to small, secreted effector-like proteins (Liu *et al.* 2012). Candidates were expressed in a yeast expression system and used for infiltration into diagnostic wheat lines. SnTox1 was identified as the protein that specifically caused necrosis in wheat lines carrying *Snn1*, with its identity confirmed using transgenic approaches in virulent and avirulent *P. nodorum* strains (Liu *et al.* 2012). After cleavage of the 17 amino acid predicted signal peptide, the mature SnTox1 protein is 100 amino acids long and contains 16 cysteine residues, a common feature among avirulence effectors (Liu *et al.* 2012).

*SnTox1* is widely prevalent in *P. nodorum* and found to be present in 85% of a global collection of 777 isolates, whereas it was absent in all isolates collected from wild grasses that are avirulent on wheat (Liu *et al.* 2012; McDonald *et al.* 2013). Although the molecular basis of necrosis-induced effector-triggered susceptibility is largely unknown, hallmarks of programmed cell death have been observed for SnTox1 in susceptible wheat, including oxidative burst, DNA laddering, and pathogenesis-related gene expression (Liu *et al.* 2012). SnTox2 is a small secreted peptide of ∼7 kDa that causes necrosis on wheat lines carrying the *Snn2* gene on chromosome 2DS (Friesen *et al.* 2007). Compatible SnTox2-*Snn2* and SnToxA-*Tsn1* interactions are additive in their effects for susceptibility (Friesen *et al.* 2007), showing that in contrast to the classical gene-for-gene model, the presence of two effector-host sensitivity gene interactions results in more disease than one interaction alone (Friesen *et al.* 2008).

In view of the complex nature of host resistance and the significant genotype by environment (G × E) interaction (Friesen *et al.* 2008), screening segregating wheat populations with *P. nodorum* effectors has the potential to rapidly increase basal levels of host resistance to both pathogens by selection of effector insensitive lines for cultivar development and to discover (and remove) the corresponding host sensitivity loci. To date, only the *Tsn1* host sensitivity locus has been cloned, with *Snn1* (Liu *et al.* 2004; Reddy *et al.* 2008), *Snn2* (Friesen *et al.* 2007), and *Snn3-B1* (Zhang *et al.* 2011) genetically mapped to chromosomes 1BS, 2DS, and 5BS, respectively. In this study, we use SnTox1 to screen a recently completed eight-parent wheat multiparent advanced generation inter-cross (MAGIC) population (Mackay *et al.* 2014) genotyped with a 90,000 feature single-nucleotide polymorphism (SNP) array, fine-mapping the *Snn1* locus to chromosome 1BS. The peak marker for *Snn1* was converted to the KASP genotyping platform, providing a cheap and flexible genetic marker for breeders and researchers.

## Materials and Methods

### Wheat germplasm and high-density genotyping

The eight-parent MAGIC population described by Mackay *et al.* (2014) was used for all phenotypic screening. Briefly, the complete population consists of >1000 lines generated from three rounds of inter-crossing between eight elite United Kingdom wheat varieties (Alchemy, Brompton, Claire, Hereward, Rialto, Robigus, Soissons, Xi19) followed by five rounds of selfing to remove heterozygosity. Here, a subset of 642 lines (see Supporting Information, Table S1) were screened for SnTox1 sensitivity. Genotype data were generated using the wheat Illumina 90,000 SNP array (Wang *et al.* 2014), as described by Mackay *et al.* (2014). Genotyping was provided as a service by the Department of Primary Industries (Victorian AgriBiosciences Center, Bundoora, Australia). SNPs were called using GenomeStudio software (Illumina) using the clusterfiles developed by Wang *et al.* (2014). SNPs for downstream analyses were selected on the basis of genotyping success rate (>0.91) and minor allele frequency (>0.05). All genotype data are available from http://www.niab.com/magic/. In total, 15,514 polymorphic markers were used for quantitative trait locus (QTL) mapping (listed in Table S2). Genotype calling for selected SNPs was checked manually with GenomeStudio. Additionally, for SNPs not included in the initial analysis due to failure to automatically call genotypes, but predicted to map to the short arms of the group 1 chromosomes from BLASTn analysis to flow sorted wheat chromosomes (http://urgi.versailles.inra.fr/; The International Wheat Genome Sequencing Consortium 2014), genotypes were called manually with GenomeStudio. Updated allelic bins were defined with manually defined x and y coordinates, allowing for more accurate calling. A subset of 12 of these markers, selected by high linkage disequilibrium (D′ >0.94 and R^2^ >0.6) to the peak marker, were included in a second QTL scan.

### SnTox1 protein production and MAGIC phenotyping

**Figure 1:**
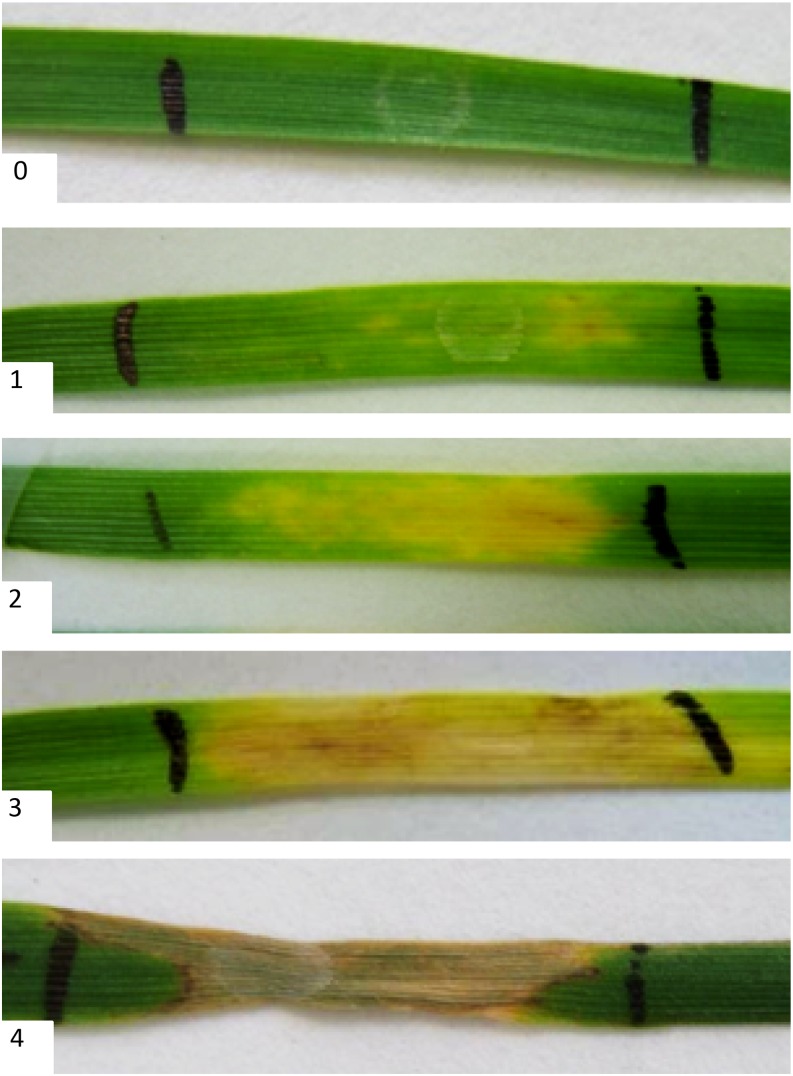
Score protocol for *P. nodorum* effector sensitivity. All scores were made on the infiltrated region delineated by indelible nontoxic marker pen as follows: 0: no visible effect, 1: very slight chlorosis, 2: fully chlorotic, 3: chlorosis and, or slight necrosis, 4: fully necrotic.

*SnTox1* was heterologously expressed in *Pichia pastoris*, as previously described (Tan *et al.* 2014). The Pichia culture filtrate containing SnTox1 desalted in 10 mM sodium phosphate buffer (pH 7), filter-steralized, used undiluted. Empty vector culture filtrate was used as a control. Effectors were stored in a refrigerator at 4° on ice until used. MAGIC lines were grown in 96-well trays filled with medium grade compost and germinated in a heated and lit glasshouse held at 20° day, 17° night with a 16-hr photoperiod. Each line was represented by three replicates. All lines and replicates were allocated well positions with a randomized design. Outer cells of each tray were sown with discard to help reduce edge effects. Seedlings were grown for 2 wk, after which the third leaf from each plant was infiltrated with SnTox1 using a 1-mL syringe without needle, and the extent of infiltration along the leaf marked with a nontoxic pen. In addition, negative water infiltration controls were included for every MAGIC line. One week after infiltration, infiltrated leaves were scored visually for necrosis based on the protocol previously described by Waters *et al.* (2011), modified to a 0 to 4 scoring scale, where 0 = complete insensitivity and 4 = extensive necrosis (Figure 1).

### Statistical analyses and bioinformatics

SnTox1 phenotypic data were analyzed using REML as implemented in GenStat version 14 (VSN International, Hemel Hempstead, UK). Line means estimated as fixed effects were used in subsequent QTL analyses. Heritability among MAGIC line means was estimated as (variance among MAGIC lines estimated as random effects) / (variance among MAGIC lines + average variance of differences among MAGIC lines). QTL analyses were undertaken as described in Mackay *et al.* (2014) using the R package lme4 (Bates *et al.* 2013). Although the MAGIC population was designed to produce a population with uniform kinship relationships, some structure remains. As the full pedigree of the population is known, this was accounted for using a mixed model with variance components to account for each stratum (between funnels and between outcrossed plants within funnels). The full MAGIC population pedigree is available from http://www.niab.com/magic/. Marker-trait associations were considered significant above the Bonferroni corrected *P* = 0.05 threshold (-log_10_*P* = 5.49). SNPs were ordered in the resulting Manhattan plots according to the consensus genetic map generated with the wheat 90k SNP chip (Wang *et al.* 2014). For three significant SNPs, genetic position on the consensus map was amended by identification of their corresponding most highly correlated SNPs using Pearson correlation coefficient. Available genomic sequence flanking each SNP were used for BLASTn analysis against rice (*Oryza sativa* L. ssp. *japonica* cv. Nipponbare) coding regions (CDS) (MSU Osa1 assembly v6.1, http://rice.plantbiology.msu.edu/), brachypodium (*Brachypodium distachyon* accession BD21) gene models (assembly v1.0, using sequence data produced by the US Department of Energy Joint Genome Institute, http://modelcop.org/, MIPS/JGI v1.2 annotation), and hexaploid wheat cv. ‘Chinese Spring’ genome survey sequence (GSS) from flow-sorted chromosome arms (http://urgi.versailles.inra.fr/; The International Wheat Genome Sequencing Consortium 2014), as described by Wang *et al.* (2014). Gene predictions were undertaken on wheat genome sequence contigs using FGENESH (Solovyev *et al.* 2006). Interspecies orthology was determined by BLAST, with an e-value threshold of <1e^-4^.

### Development of KASP markers

Selected SNPs were converted to the Kompetitive Allele-Specific PCR (KASP) genotyping platform (LGC Genomics), based on single-plex technology that employs a universal fluorescent reporting system in conjunction with allele-specific primers. SNP flanking sequences (Wang *et al.* 2014) were used for BLASTn searches of the genome survey sequence for wheat cultivar ‘Chinese Spring’ (The International Wheat Genome Sequencing Consortium 2014; available via http://wheat-urgi.versailles.inra.fr/). To identify putative homeologous copies on the A, B, and D wheat subgenomes, the highest matching sequence contigs were used as inputs for gene predictions using FGENESH (http://www.softberry.com/) using the ‘*Triticum aestivum*’ model. Predicted gene models for each homeologous set were aligned with ClustalW (Thompson *et al.* 1994) and manually edited with GENEDOC v2.6 (http://www.nrbsc.org/gfx/genedoc/). Homeologue-specific nucleotides flanking each targeted SNP were highlighted for incorporation into KASP primer design. Genomic DNA from the eight MAGIC parents and a subset of the progeny (see Table S1) was extracted using a modified Tanksley protocol (Fulton *et al.* 1995). DNA concentrations were determined using a Nanodrop 200 spectrophotometer (Thermo Scientific), and diluted to a final concentration of 10 ng/µL with sterile distilled water. KASP genotype data were returned from the service provider as .csv files, analyzed with SNP Viewer v.1.99 (http://lgcgenomics.com/), and compared with the corresponding SNP calls from the 90,000 SNP array.

### Data availability

MAGIC germplasm is available from NIAB on request. ISelect 90k SNP genotype data is available from NIAB online at www.niab.com/magic/. SnTox1 sensitivity scores for the MAGIC lines screened are available in Table S1. The genetic markers used for QTL mapping, and their genetic map positions, are listed in Table S2.

## Results

### SnTox1 phenotyping

SnTox1 sensitivity was assessed on 642 MAGIC lines, with line means adjusted for block and position effects (see Table S1 and Figure 1). Parental lines exhibited varying SnTox1 sensitivity, with adjusted mean values ranging from 0.03 (Rialto) to 2.36 (Soissons). Analysis of progeny sensitivities identified transgressive segregation, with values ranging from -0.37 to 4.00, and a mean of 1.47. SnTox1 sensitivity of line means was found to be highly heritable, at 56.0%.

### QTL mapping of SnTox1 sensitivity

**Figure 2:**
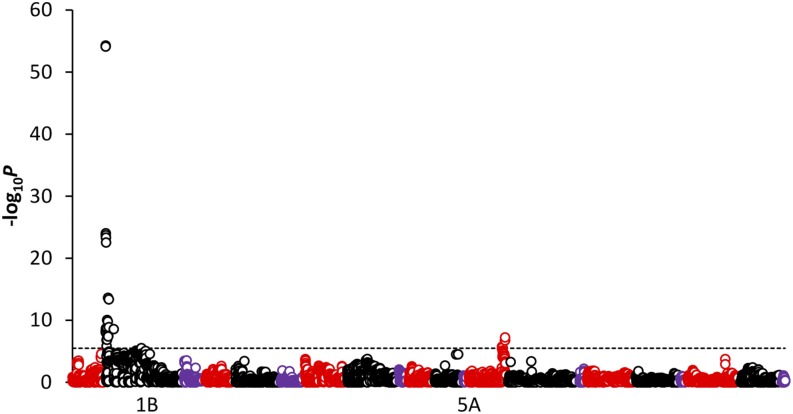
Identification of SnTox1 sensitivity quantitative trait locus in the multiparent advanced generation inter-cross (*i.e.*, MAGIC) population. Significant single-nucleotide polymorphism associations (-log_10_*P* ≥ 5.49, indicated by the dashed line) were identified on chromosomes 1B and 5A. Unmapped markers are not shown. Additional markers identified in the region by manual re-scoring (see *Materials and Methods*) and used in a second association scan are not shown here, but are listed in Table 1.

**Figure 3:**
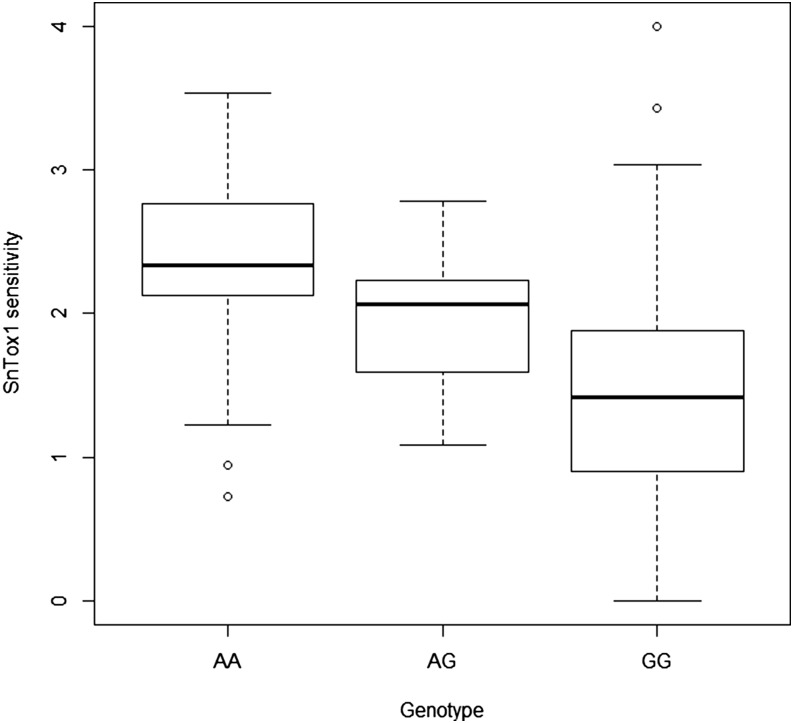
Box plot for peak marker Excalibur_c21898_1423 on chromosome 1BS, contrasting allelic state (homozygous AA, heterozygous AG, and homozygous GG genotypes) with SnTox1 sensitivity (y-axis, adjusted means rescaled to a 0 to 4 scale).

Using 15,514 SNPs across 642 MAGIC lines, QTL mapping of SnTox1 sensitivity in the MAGIC population identified 42 significant (-log10*P* > 5.49) SNPs (Table 1, Figure 2, and File S1). Of these, 29 genetically mapped to chromosome 1B, 11 to 5A, and 2 were unassigned. By far the strongest QTL was located on chromosome 1B, with the highest marker-trait associations identified for SNPs Excalibur_c21898_1423 (-log10*P* = 55.29) and BS00093078_51 (-log10*P* = 54.12), which cosegregated at 8.36 cM. The peak marker(s) explained 25.9% of the variation in line means for SnTox1 sensitivity, with box-plots of genotype at Excalibur_c21898_1423 *vs.* host sensitivity clearly indicating phenotypic partitioning according to genotype (Figure 3). The second genetic locus was identified by SNP BS00068108_51 (-log10*P* = 7.25) on the long arm of chromosome 5A, termed here *QSnn.niab-5A.1* following Graingenes annotation (www.graingenes.org/), and explained 4.1% of the variation. Modeled together, the two peak SNPs at *Snn1* and *QSnn.niab-5A.1* explained 28.7% of the variation in line means. Of the six significant SNPs that lacked a genetic map position in the consensus map, all but BS00022296_51 could be tentatively assigned to chromosome arms on the basis of high linkage disequilibrium with the peak *Snn1* marker (D′ > 0.94) or by BLASTn hits to wheat genome survey sequence generated from flow-sorted chromosome arms (Table 1). To enrich the 1BS QTL with additional markers, previously uncalled or unusable (due to quality control parameters) SNPs predicted to map to the short arm of the group 1 chromosomes by BLASTn analysis to the wheat reference genome sequence were recalled manually. Twelve markers in high linkage disequilibrium (D′ >0.94, R^2^ >0.6) with the peak markers (Excalibur_c21898_1423, BS00093078_51) were recovered: BobWhite_c4303_524, BS00011824_51, BS00015608_51, BS00022296_51, BS00026180_51a, BS00030768_51, BS00071333_51, BS00076192_51b, BS00111170_51b, Excalibur_c35316_388a, Jagger_c5878_119, and Kukri_c37738_417. Inclusion of these additional SNPs within subsequent QTL analyses (total number of markers = 15,526) found them to be highly significant (-log_10_*P* ≥ 42.26), although not more significant than the two best markers identified from the automated 90k SNP chip calls.

**Table 1:** Wheat genetic markers significantly associated (-log_10_*P* ≥ 5.49) with TOX1 sensitivity in the eight-parent MAGIC population (total number of significant SNPs = 54)

Marker Name	Sig.-log_10_ *P*	Ta chr, cM*^a^*	Rice Homolog*^b^*	% ID*^b^*	Brachy Homolog*^c^*	% ID*^c^*	Ta GSS BLAST Hit*^d^*	% ID*^d^*	Gene Models*^e^*
Excalibur_c21898_1423	55.29	1B, 8.361	Os02g18940 (**Os05g01040**)	89	(**2g40070**)	.	1BS_3482116	100	3
BS00093078_51	54.12	1B, 8.361	**Os05g01090** (**Os05g01110**)	73	**2g40120** (**2g40040**)	78	1BS_3479333	99	3
BS00026180_51a*^f^*	51.61	1B, 8.361	.	.	.	.	1BS_3448531	98	1
IAAV5782	38.60	U	Os11g44580	68	.	.	**1BS_3443196**	100	1
Kukri_c37738_417*^f^*	38.44	1B, 9.679	.	.	.	.	1DS_108919	96	
BobWhite_c4303_524*^f^*	38.18	1B, 9.679*^g^*	**Os05g01250**	94	**2g39980**	97	1DS_1882757	99	
BS00011824_51*^f^*	37.87	U	.	.	**2g40046**	92	1DS_1907090	100	
BS00030768_51*^f^*	37.81	1B, 19.79	.	.	.	.	1BS_345816	99	3
BS00022296_51*^f^*	37.27	U	.	.	**2g39247**	.	**1AS_1838762**	100	1
BS00076192_51b*^f^*	35.50	1B, 60.62	.	.	4g09944	.	1BS_3456702	100	2
BS00111170_51b*^f^*	35.29	1B, 9.68	.	.	.	.	**1BS_3454955**	99	1
Excalibur_c22958_433	24.95	1B, 8.361*^g^*	.	.	.	.	2BL_8088363	99	
Jagger_c5878_119*^f^*	24.19	1B, 8.361	**Os05g01060** (**Os05g10150**)	85	**2g40100** (**2g40090**)	85	1BS_3451992	100	3
Kukri_c44369_131	24.04	1B, 8.361	Os02g18940 (**Os05g01040**)	93	**2g40077**	89	1BS_3482116	100	3
BS00071333_51*^f^*	24.02	1B, 8.361	.	.	.	.	**1BS_2548463**	99	1
Excalibur_c35316_388a*^f^*	24.02	U	.	.	2g01490	85	1BS_3478312	99	2
BS00015608_51*^f^*	24.02	U	.	.	1g44420	86	1AS_3265927	100	1
BS00022504_51	23.73	1B, 8.361	(**Os05g01230 Os05g01240**)	.	(2g39980*)	.	1BS_3475480	99	7
RAC875_c24163_155	23.32	1B, 8.361	Os04g30200	80	.	.	3DL_6837169	96	
BS00082565_51	22.55	1B, 9.679	(**Os05g01280**)	.	(**2g03650**)	.	1BS_3483503	99	4
TA003422-0757	14.03	1B, 41.057	.	.	.	.	**1BS_3464287**	100	3
BS00011695_51	13.63	1B, 41.057	(**Os05g01780, Os05g01790**)	.	**2g39347** (**2g39340**)	70	1BS_3440902	99	3
BobWhite_c14271_1379	13.39	1B, 43.858	.	.	.	.	5BS_1482084	99	
Excalibur_c16851_835	10.05	1B, 31.04	Os05g51630	90	**2g39230**	92	1BS_3480523	99	7
IAAV4194	9.83	1B, 31.094	**Os05g02010**	92	**2g39150** (**2g38750**)	91	1BS_3479684	100	2
BS00071161_51	8.92	1B, 43.858	(**Os05g01020**)	.	(**2g40046**)	.	1BS_3450821	99	6
BS00022429_51	8.77	1B, 30.338	.	.	4g14110	80	**1BS_3484197**	99	7
Excalibur_c10657_796	8.70	1B, 8.361	**Os05g01060**	89	**2g40100**	92	1DS_1904229	100	
BS00022180_51	8.60	1B, 43.858*^g^*	(**Os05g01090**, **Os05g01110**)	.	**2g40120** (**2g40040**)	74	1BS_3479333	99	3
BobWhite_c5793_372*^h^*	8.36	1B, 9.679	Os10g02990	.	.	.	1BS_1682563	99	1
BS00050522_51	8.11	1B, 5.275	(**Os05g01030**)	.	.	.	1BS_3482114	99	2
BS00022482_51	8.04	1B, 9.679	(**Os05g01280**)	.	.	.	1BS_3483503	99	4
Kukri_rep_c106834_139	8.04	1B, 9.679	(**Os05g01230 Os05g01240**)	84	(**2g39990**, **2g40000**)	.	1BS_3475480	98	7
BS00022505_51	7.90	1B, 9.679	(**Os05g01230 Os05g01240**)	86	**2g39990** (**2g40000**)	86	1BS_3475480	99	7
BS00067961_51*^i^*	7.79	U	.	.	4g05910	77	1BS_3432531	99	2
Tdurum_contig47083_278	7.51	1B, 41.213	(**Os05g01550**)	97	**2g39700**	74	1BS_3435018	98	5
BS00070706_51	7.39	1B, 41.213	(**Os05g01550**)	.	(**2g39700**)	.	1BS_3435018	99	5
BS00068108_51	7.25	5A, 144.263	.	.	.	.	5AL_1712099	99	
CAP7_c3299_342	6.88	1B, 18.741	.	.	.	.	1AS_1937927	97	
BS00105846_51	6.87	1B, 28.761	**Os05g01710**	91	5g01710	91	1BS_3398629	99	4
Excalibur_c42255_425	6.79	5A, 144.137	.	85	3g09740	82	5AL_2807342	100	
Excalibur_c77910_272	6.79	5A, 144.137	.	.	.	.	5AL_2685434	99	
CAP7_c3299_316	6.78	1B, 18.741	.	.	.	.	1AS_1937927	97	
Excalibur_c30569_384	5.86	1B, 21.045	.	.	.	.	**1BS_3484442**	99	7
wsnp_Ku_rep_c71232_70948744	5.73	5A, 125.683	Os03g02970	88	1g77087	95	5AL_2752914	99	
Kukri_c75091_220	5.73	5A, 125.682	.	.	.	.	5AL_2752914	100	
RAC875_c32639_395	5.73	5A, 125.682	Os03g02980	87	1g77080	77	5AL_2670848	100	
wsnp_Ex_c23795_33033150	5.73	5A, 125.682	Os03g02970	86	1g77087	90	5AL_2752914	100	
wsnp_Ex_c23795_33033959	5.73	5A, 125.682	Os03g02970	86	1g77087	93	5AL_2752914	99	
Kukri_c75091_154	5.73	5A, 125.682	.	.	.	.	5AL_2752914	99	
Tdurum_contig78972_316	5.68	U	(**Os05g02020**)	.	(**2g38970**)	.	1BS_3460466	98	1
BS00022299_51	5.63	5A, 124.588	.	.	.	.	.	.	
wsnp_Ex_c23795_33033010	5.61	5A, 125.682	Os03g02970	86	1g60100	82	5AL_2752914	100	
RAC875_rep_c70803_1442	5.53	1B, 66.0715	Os10g36060	89	3g30277	93	1BL_3907000	100	1

Both mapped and unmapped markers are included. MAGIC, multiparent advanced generation inter-cross; SNP, single-nucleotide polymorphism; Sig, significance; Ta, *Triticum aestivum*; Chr, chromosome; CDS, coding sequence; GSS, genome survey sequence; EST, expressed sequence tag, LD, linkage disequilibrium.

a Wheat genetic map position according to Wang *et al.* (2014).

b Best rice homolog, based on BLASTn searches of flanking SNP sequence *vs.* rice CDS. Rice genes agreeing with established colinearity with wheat chromosome 1B are indicated in bold. Genes in parentheses list colinear rice orthologs of predicted wheat gene models identified on wheat 1BS GSS contigs. Percentage identity (% ID) indicated.

c Best brachypodium homolog, based on BLASTn searches of flanking SNP sequence *vs.* brachypodium CDS. Brachypodium genes agreeing with established colinearity with wheat chromosome 1B are indicated in bold. Genes in parentheses list colinear brachypodium orthologs of predicted wheat gene models identified on wheat 1BS GSS contigs. All brachypodium genes are prefixed by “Bradi.”

d Best hit to wheat genome sequence, based on BLASTn searches of flanking SNP sequence *vs.* wheat GSS of flow-sorted chromosomes.

e Number of FGENESH predicted genes on wheat GSS contig.

f Re-called SNPs in high LD with Excalibur_c21898_1423, as measured by R^2^ (>0.6), D1 (>0.94).

g Genetic map position amended (see File S1).

h Marker from the same gene found by Reddy *et al.* (2008) to be proximal to *Snn1* (based on EST BF293222). Wheat contigs predicted to possess NBS-LRR genes are highlighted in bold.

i Classified as unmapped here, see Text S1 in File S1.

To attempt to identify additional genetic loci controlling SnTox1 sensitivity, QTL analyses were repeated with the peak 1B marker (Excalibur_c21898_1423) as a covariate. These analyses failed to identify any additional QTL (Figure S1), indicating only two major genetic loci controlling SnTox1 sensitivity were segregating in the MAGIC population. It is of course possible that additional loci with smaller effects were segregating, but were not detected.

### Comparative analysis of the Snn1 region

**Figure 4:**
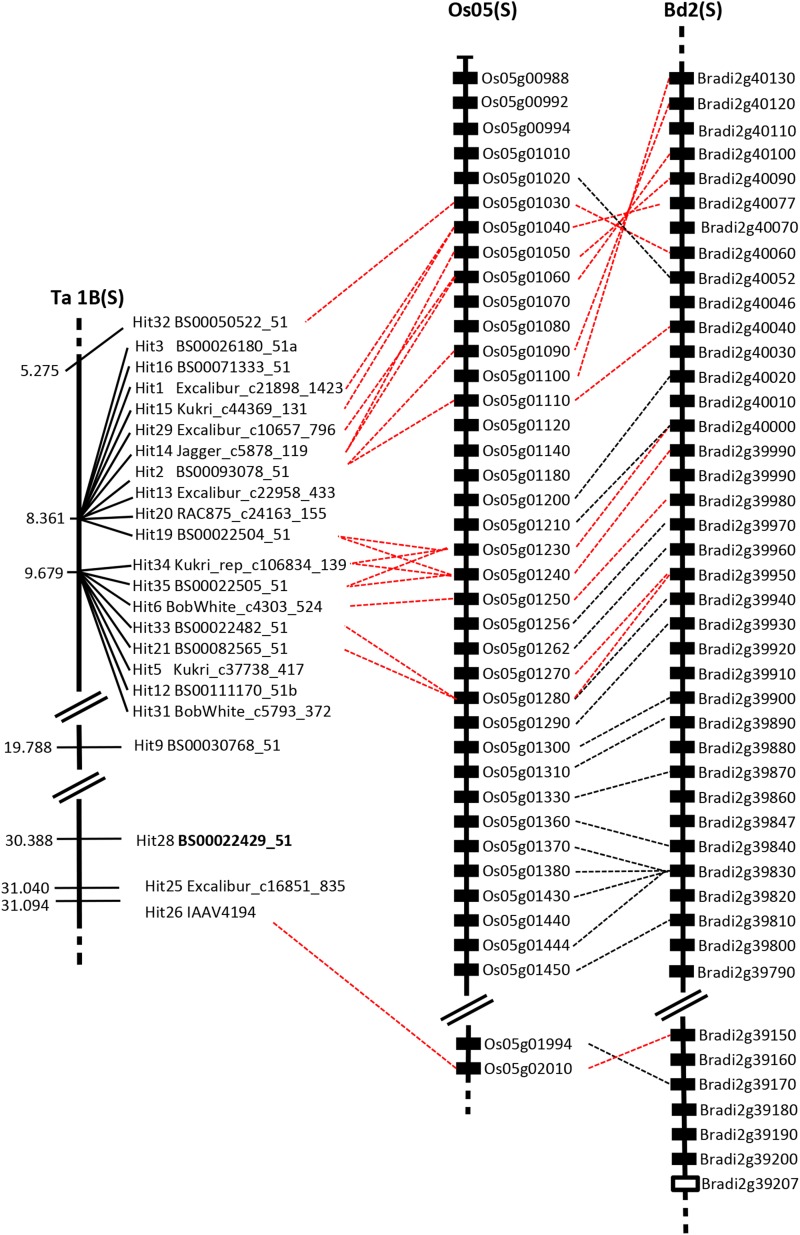
Significant wheat genetic markers (between 0 and 9.7 cM on the short arm of chromosome 1B), and colinearity with the physical maps of rice and brachypodium. Red dashed lines link wheat genes with ortholgous in rice and brachypodium. Black dashed lines link rice and brachypodium orthologs. Brachypodium NBS-LRR genes are indicated in white. The wheat SNP located on a sequence contig predicted to contain an NBS-LRR gene is highlighted in bold. Chromosome arm is indicated: S (short), L (long).

To investigate the possible gene content within the *Snn1* region, markers mapped to the region were used to identify the region of the short arm of rice chromosome Os05, and long arm of brachypodium chromosome 4, known to display macro-colinearity with wheat 1BS (Peng *et al.* 2004; International Brachypodium Initiative 2010). *Snn1* previously has been mapped to a 4.7-cM interval between restriction fragment length polymorphism marker *ksuD14* and the 1BS telomere (Liu *et al.* 2004; 2012). *KsuD14* lies within a sequenced wheat contig on the short arm of the 1D homeologous chromosome (GenBank accession AF532104S1), predicted to contain four genes, including the cloned resistance gene *Lr21* (Huang *et al.* 2004). However, BLASTn analysis of the four predicted CDS to rice did not identify orthologous genes on the colinear region of rice chromosome Os05. Genomic sequence flanking the *Snn1* region SNPs identified here were used as BLASTn queries against all rice and brachypodium gene models (Table 1). To further aid comparative analyses, predicted gene content of wheat GSS contigs identified as possessing *Snn1* region SNPs were determined, and the resulting gene models used to identify rice and brachypodium orthologs (Table 1). In total, 19 wheat SNPs/contigs identified the following orthologs in the two sequenced grass species investigated: (1) Rice: 16 orthologous rice genes in the colinear region of Os05, spanning a region from LOC_Os05g02010 (based on orthology with wheat marker IAAV4194) to the short arm telomere. This delimited a physical region of 574 kb in rice, containing 84 predicted genes and 21 transposable elements (see Table S3). No NBS-LRR genes were present in this rice interval nor were any genes homologous to the cloned wheat SnToxA effector sensitivity locus, *Tsn1*. (2) Brachypodium: 16 orthologous brachypodium genes in the colinear region of Bradi2g, spanning a region from Bradi2g38970 to Bradi2g40120 (orthologous to wheat SNPs IAAV4194 and BS00093078_51, respectively). This delimited a physical interval of 884 kb in brachypodium, predicted to contain 118 genes. No genes homologous to *Tsn1* were identified in this region. Although two NBS-LRR genes were found (Bradi2g38987, Bradi2g39207), the use of CDS from these gene as a queries for BLASTn searches of the wheat genome identified best hits on chromosomes 1D (IWGSC_GSS_1899471) and 7A (IWGSC_GSS_4256951), respectively. No NBS-LRR genes were found in brachypodium within the region colinear with the highest associated wheat SNPs (hits 1 to 21, see Figure 4). In wheat, predicted NBS-LRR genes were identified on wheat GSS contigs 1BS_3464287 (anchored by SNP TA003422-0757, 1BS at 41.057 cM), 1BS_3484197 (SNP BS00022429_51, 1BS at 30.338 cM), 1BS_3443196 (SNP IAAV5782, 1BS based on BLASTn of wheat GSS), 1AS_1838762 (SNP BS00022296_51, unmapped but allocated to group 1 chromosomes by BLAST), 1BS_3454955 (SNP BS00111170_51b, 1BS at 9.68 cM), 1BS_2548463 (SNP BS00071333_51, 1BS at 8.361 cM), and 1BS_3484442 (Excalibur_c30569_384, 1BS 1t 5.86 cM) (Table 1). Two additional NBS-LRR genes have been reported as being located in the *Snn1* region (BE498831 and BF474204) (Reddy *et al.* 2008), which correspond to GSS contigs 1BS_3440935 and 1BS_ 3412727, respectively. However, none of the SNPs on the 90k array for which genotype data were returned originated from these contigs, indicating at least nine NBS-LRR genes are located within the region.

### Development of KASP markers for Snn1

**Figure 5:**
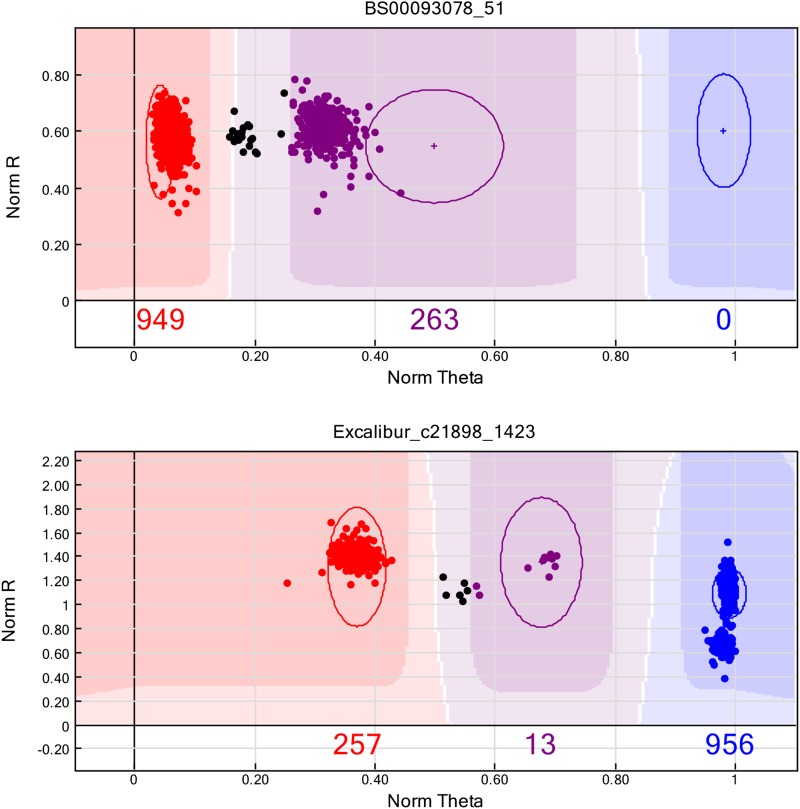
Allele calling for the two peak *Snn1* SNPs in the 90k SNP dataset, using GenomeStudio software. SNPs BS00093078_51 (top) and Excalibur_c21898_1423 (bottom) were classified as dominant and codominant, respectively, following the standard calling procedures. Visual inspection of BS00093078_51 shows clustering of AA homozygotes (red) and AB heterozygotes (purple), ‘no call’ genotypes (black) located between the AA and AB clusters, and an absence of BB genotypes. These ‘no call’ genotypes represent true heterozygotes, based on the observation that both the BB and AB clouds have two sub-clusters.

Markers BS00093078_51 (classified as dominant using the default SNP calling parameters) and Excalibur_c21898_1423 (codominant) were found to be highly significant (-log_10_*P* = 55.29 and 54.12, respectively). However, manual inspection of SNP calls showed that BS00093078_51 to possess a cluster of heterozygotes, expected in this MAGIC population, as some heterozygosity will remain, depending on how advanced recombinant inbred line development was at the time on genotyping (Figure 5). This suggests confounding of allele calling in the original panel of wheat accessions used to define the calling parameters for the SNP chip, possibly due to additional amplification from non-target homeologues. Thus, visual inspection indicates that BS00093078_51 is more accurately classified as a co-dominant marker. SNP Excalibur_c21898_1423 shows the clustering pattern expected from a co-dominant marker in the absence of amplification from non-target homeologues. Accordingly, both SNPs were selected for attempted conversion to KASP markers. BS00093078_51 was found to be transferable to the KASP system, and was validated on a panel of 95 wheat varieties (see Figure S2 and Table S4).

## Discussion

### The use of effectors *vs.* genetic markers for disease resistance breeding

The *P. nodorum*-wheat pathosystem was until recently thought to be due to the interaction of a suite of nonspecific toxins and cell wall degrading enzymes (Solomon *et al.* 2006; Liu *et al.* 2009). Wheat resistance to *P. nodorum* was quantitative, and controlled largely by numerous, weak and environment-specific QTL (Xu *et al.* 2004). The recent identification of necrotrophic effectors in *P. nodorum* provides tools with which to break down host resistance into its constituent parts, and so help study the disease and help develop resistant varieties. *P. nodorum* effectors have already been used by breeders to screen for insensitive germplasm within their breeding programs (Tan *et al.* 2014), resulting in an estimated saving of AUD $50m in Australia alone. However, the ability to develop genetic markers diagnostic for allelic state at the corresponding host sensitivity loci provides additional benefits. First, it allows workflows to be streamlined, as wheat breeders now routinely use genetic markers, allowing host sensitivity markers to be easily incorporated into current practices. Second, as chromosomal regions in close physical linkage with host sensitivity loci may harbor undesired allelic variants, the use of genetic markers allow precise manipulation of other loci on the same chromosome arm. Ultimately, the identification of the genes underlying host sensitivity loci will allow molecular and biochemical characterization of host-pathogen interactions. Such knowledge will help advance the pace of genomics-informed disease resistance breeding in wheat and other crops.

### Genetic mapping of sensitivity to SnTox1 using MAGIC

The hexaploid wheat genome is large and complex (2*n* = 6X = 42, total size = 16 GB), complicating fine-scale genetic analyses. However, numerous genomic tools are now emerging in wheat, including genome survey sequence and dense SNP chips, as used in this study. In addition, recent developments in genetic mapping population design widen the opportunities for genetic analyses in crops. MAGIC is one of a number of multi-parent population designs now emerging in plant sciences (see the recent Multiparent Populations Collection in the journals *Genetics* and *G3*; http://www.genetics.org/site/misc/multiparental_populations.xhtml). The benefit of such populations is that they allow the use of linkage and association genetic methodologies for genetic analysis, while avoiding the difficulties imposed by highly structured association mapping populations (Mackay and Powell 2007). MAGIC populations are ideally suited to overcome many of the current barriers to genetic trait dissection in wheat, due to the high levels of genetic diversity captured (via multiple founders) and genetic recombination (via multiple rounds of inter-crossing) (Cavanagh *et al.* 2008). To date, only two wheat MAGIC populations are publicly available: the eight-parent winter United Kingdom wheat (Mackay *et al.* 2014) and the four-parent spring Australian wheat (Huang *et al.* 2012) populations. Here we use the United Kingdom MAGIC population genotyped with a 90,000 feature SNP array to fine-map the wheat *Snn1* locus. Adjusted mean SnTox1 sensitivity in the parental lines ranged from 0.03 (Rialto), to 2.36 (Xi19). Genotypic scores at the three most significant SNPs discriminate between Xi19 and Soissions on the one hand, and the other six founders on the other (Table S5). We also identified SNPs uniquely tagging Soissions (Excalibur_c10657_796) and uniquely tagging Xi19 (Kukri_c37738_417), which map within a 1.3-cM interval. Both of these are highly significant (-log_10_*P* ≥ 8.11), with their joint effects accounting for a similar proportion of the total sum of squares (SS) compared with the peak SNP, Excalibur_c21898_1423 (29% compared to 26%). Therefore, SnTox1 sensitivity is introduced in the population via Xi19 and Soissons. The Xi19 QTL is the larger effect, accounting for 20% of the SS. Given the previous reports of a single major genetic locus, *Snn1*, controlling SnTox1 sensitivity at this location (Liu *et al.* 2012; Reddy *et al.* 2008), it is likely to represent a single genetic locus. However, in the absence of further information we cannot unambiguously state that these are independent alleles at the same QTL.

In addition to *Snn1*, we identified a second SnTox1 sensitivity locus on chromosome 5A. Previous analysis of SnTox1 and culture filtrates from SnTox1 knock out mutants has identified a sensitivity locus within a >10-cM interval on chromosome 4B in the ITMI bi-parental mapping population (Liu *et al.* 2012). However, no 4B QTL was identified in this study. Similarly, the 5A QTL *QSnn.niab-5A.1* identified here has not been previously reported. Although the reasons for these differences could be numerous, it is most likely due to the different nature of the germplasm resources used: the ITMI population was constructed from a cross between a Mexican spring variety and a synthetic hexaploid wheat (Van Deynze *et al.* 1995), whereas elite winter-sown United Kingdom germplasm was used to construct the MAGIC population investigated here.

### Comparative mapping of the Snn1 locus

Although genome survey sequence is available, wheat does not contain a completed physical map. This is largely due to the extremely large size of the wheat genome (16 Gb) in comparison to other sequenced cereal species (*e.g.*, rice 430 Mb, brachypodium 255 Mb). However, conservation of gene content and order between members of the Poaceae has meant that colinearity is commonly used as a tool for inferring possible gene content in wheat and other large genome cereal species (*e.g.*, Cockram *et al.* 2010a,b). Colinearity between wheat 1BS and rice (Os05g) and brachypodium (Bd2) was found to be variable across the *Snn1* locus. Nevertheless, based either on homology of the gene in which the genotyped SNP originated, or on additional genes on the same genomic survey sequence contig as the SNP, 16 of the 32 most significant markers possessed orthologs on the colinear regions of the rice genome, with 18 possessing orthologous brachypodium genes. Colinearity between rice and brachypodium showed good overall levels of microcolinearity. In the regions colinear with the most significant wheat SNPs and associated contigs (between 5.275 and 8.361 cM), 62% of rice genes were conserved in brachypodium, whereas 68% of brachypodium genes were conserved in rice. Additionally, a small local inversion between these two species was observed, predicted to involve the wheat region carrying the peak SNP. We note that the rice region involved is within 60 kb of the chromosome Os05 short arm telomere, indicating this rearrangement could have occurred as a result of the macrochromosomal rearrangements that resulted in different chromosome numbers between rice and brachypodium. Thus, while colinearity with sequenced grass species will clearly be of use in map-based cloning of *Snn1*, differences in gene content mean that sequenced physical wheat maps of the region for cultivars showing SnTox1 sensitivity will be essential. This was found to be the case in the cloning of *Tsn1*, which is encoded by a gene absent from the colinear region of rice and other sequenced cereal species (Faris *et al.* 2010). We note that SNP marker IAAV5782 (-log_10_*P* = 38.60) originates from the same wheat contig as a marker (BE498831) previously shown to cosegregate with *Snn1* (Reddy *et al.* 2008). As the peak SNP identified in this study was far more highly associated than IAAV5782 (-log_10_*P =* 55.29 *vs.* 38.60, respectively), this illustrates the additional power available in the MAGIC population, despite it not having been constructed specifically to investigate SnTox1 sensitivity.

### Effector sensitivity *vs.* adult plant susceptibility

SNB is a complex disease. Although sensitivity to SnToxA has been shown to be a strong predictor of field SNB incidence, poor correlation between SnTox1 and SnTox3 sensitivity and disease susceptibility has previously been reported (Waters *et al.* 2011; Oliver *et al.* 2009). We have hypothesized that there are many other undiscovered effectors, and that interactions between these in establishing SNB have not been fully documented (Tan *et al.* 2014). Despite this, the role of *Snn1* in conferring resistance SNB recently has been confirmed at the seedling and adult stages (Phan *et al.* 2015). Infection of a double haploid wheat mapping population segregating for *Snn1* with *P. nodorum* SN15 identified *Snn1* as a major SNB QTL at both growth stages, based on the severity of tissue necrosis. Furthermore, *Snn1* was also confirmed as a major SnTox1 sensitivity QTL in the same population. These results demonstrate that the SnTox1-*Snn1* interaction contributes to SNB disease incidence both at seedling and adult stages, and illustrate the utility of developing and using *Snn1* linked markers for marker assisted selection.

### Diagnostic markers for Snn1

Misclassification of SNP marker BS00093078_51 as dominant using the standard SNP calling protocol was likely due to a combination of the following reasons: (1) coamplification occurred from one or more of the nontarget homeologues in the MAGIC lines; (2) allele bin creation was constructed using a training set composed of a large and diverse set of germplasm, including varieties from around the world, as well as synthetic, tetraploid and diploid wheats. Thus, where nontarget homeologues were not amplified in the training set, a SNP allele calling bin would have been created (in this example allele call BB). In such instances, the heterozygote bin would include heterozygotes (AB), as well as homozygotes + nontarget homeologue (BBBB) and heterozygotes + nontarget homeologue (ABBB). Thus, allele calling would be confounded, as was observed here. This observation has important implications for all those using the array, as it will increase the number and accuracy of markers available for genetic and genomic studies. Indeed, marker enrichment around the *Snn1* locus was found to be possible in this study following such an approach.

KASP has become the platform of choice for wheat breeding companies, and is in common use in the cereal research community (*e.g.*, Cockram *et al.* 2012; Lister *et al.* 2013; Mackay *et al.* 2014). Only one (BS00093078_5) of the two *Snn1* SNPs successfully converted to the KASP platform, due to a combination of the need to incorporate homeologue-specific nucleotides in the primers, and the constraints of effective primer design. The successfully converted KASP marker was shown to be co-dominant, allowing discrimination of heterozygotes from homozygotes, an important feature for effective use in wheat breeding and research. The development of co-dominant KASP markers closely linked to *Snn1* provides a cheap, flexible and robust way for selecting for SnTox1 insensitivity in wheat germplasm.

## 

## Supplementary Material

Supporting Information
