# Supplementary material for: Fine-Mapping the Wheat Snn1 Locus Conferring Sensitivity to the Parastagonospora nodorum Necrotrophic Effector SnTox1 Using an Eight Founder Multiparent Advanced Generation Inter-Cross Population
Source: G3 (Bethesda). 2015 Sep 24;5(11):2257–66. doi: 10.1534/g3.115.021584 (PMC4632045; doi:10.1534/g3.115.021584)
Supplement: Supporting Information [file supp_5_11_2257__index.html]

Fine-Mapping the Wheat Snn1 Locus Conferring Sensitivity to the Parastagonospora nodorum Necrotrophic Effector SnTox1 Using an Eight Founder Multiparent Advanced Generation Inter-Cross Population — Supporting Information 

# Fine-Mapping the Wheat *Snn1* Locus Conferring Sensitivity to the *Parastagonospora nodorum* Necrotrophic Effector SnTox1 Using an Eight Founder Multiparent Advanced Generation Inter-Cross Population

## Supporting Information for Cockram *et al.*, 2015

**Files in this Data Supplement:**

- Supporting Information - Figures S1-S2, Tables S1-S5, and File S1 (PDF, 282 KB)
- Figure S1 - QTL analysis for SnTox1 sensitivity, after inclusion of the peak 1B marker 'Excalibur\_c21898\_1423' as a covariate. (PDF, 176 KB)
- Figure S2 - Conversion of SNP BS00093078\_51 to the KASP genotyping platform. (PDF, 119 KB)
- Table S1 - MAGIC lines screened for SnTox1 sensitivity. (PDF, 131 KB)
- Table S3 - Predicted rice genes within the physical region colinear with the wheat *Snn1* locus. (PDF, 148 KB)
- Table S4 - Wheat lines used to validate the KASP marker for SNP Excalibur\_c21898\_1423. (PDF, 115 KB)
- Table S5 - Parental genotypic calls for significant genetic markers that co-segregate with the peak marker, Excalibur\_c21898\_1423. (PDF, 131 KB)
- File S1 - Supporting Text (PDF, 114 KB)
- Table S2 - Genetic markers used for genetic mapping. (.xlsx, 421 KB)
