# Supplementary material for: Fine-Mapping the Wheat Snn1 Locus Conferring Sensitivity to the Parastagonospora nodorum Necrotrophic Effector SnTox1 Using an Eight Founder Multiparent Advanced Generation Inter-Cross Population
Source: G3 (Bethesda). 2015 Sep 24;5(11):2257–66. doi: 10.1534/g3.115.021584 (PMC4632045; doi:10.1534/g3.115.021584)
Supplement: Supporting Information [file supp_g3.115.021584_021584SI.pdf]

G3·Genes | Genomes | Genetics

Fine-mapping the wheat *Snn1* locus conferring insensitivity to the *Parastagonospora nodorum* necrotrophic effector SnTox1 using an eight founder multi-parent advanced generation intercross population

James Cockram[*], Alice Scuderi[*§], Toby Barber[*], Eiko Furuki[†], Keith A. Gardner[*], Nick Gosman[*1], Radoslaw Kowalczyk[*‡], Huyen P. Phan[†], Gemma A. Rose[*], Kar-Chun Tan[†], Richard P. Oliver[†], Ian J. Mackay[*]

* John Bingham Laboratory, National Institute of Agricultural Botany (NIAB), Huntington Road, Cambridge, CB3 0LE, UK

§ SCIFAR Department, University of Messina, Messina, Sicily, Italy

† Centre for Crop Disease Management, Curtin University, WA6845, Australia

‡ Faculty of Life Sciences, University of Manchester, Manchester, M13 9PL, UK

[1] Current address: Bayer Crop Science NV, Ghent, Belgium

Corresponding author: NIAB, Huntington Road, Cambridge, CB3 0LE, UK. Email: james.cockram@niab.com

DOI: 10.1534/g3.115.021584

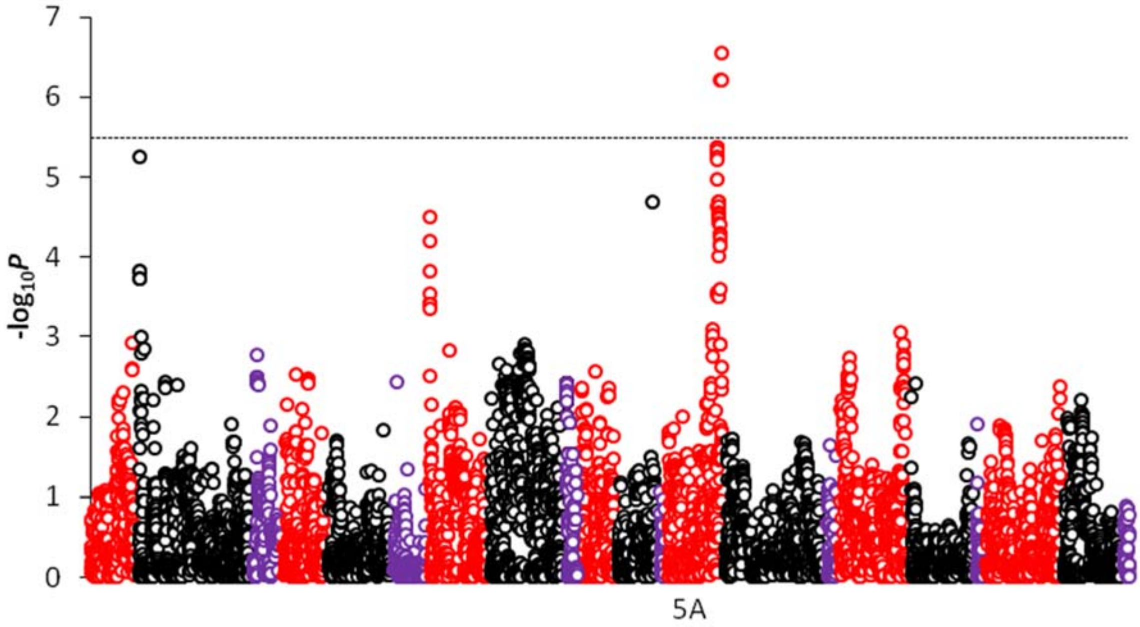

Figure S1 QTL analysis for SnTox1 sensitivity, after inclusion of the peak 1B marker 'Excalibur_c21898_1423' as a covariate. SNPs mapping to the 21 wheat chromosomes are indicated in red (A genome), black (B genome) and purple (D genome). The Bonfferoni corrected $P = 0.05$ significance threshold is indicated (dashed line). Unmapped markers are not shown.

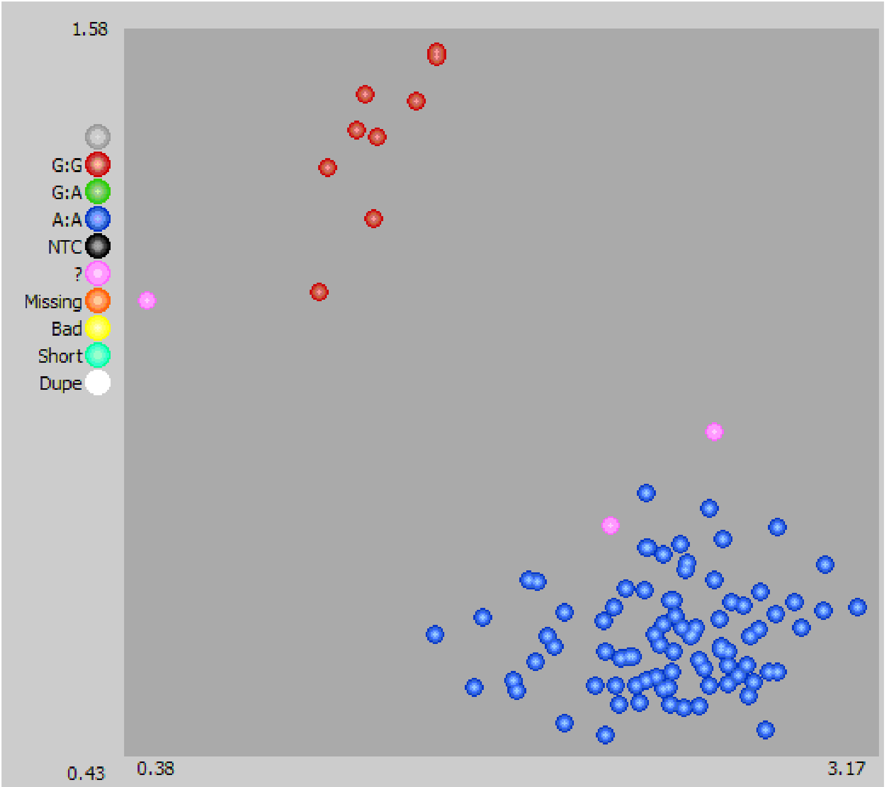

Figure S2 Conversion of SNP BS00093078_51 to the KASP genotyping platform. The assay was validated using a panel of 96 wheat varieties listed in Supplementary Table 3. Note, the KASP marker assays polymorphism on the opposite strand relative to the marker on the iSelect 90k array.

Table S1 MAGIC lines screened for SnTox1 sensitivity. Adjusted phenotypic means listed.

Variety	Funnel	Plant	Line	Mean Tox1 score
MEL_001-1b	1	1	b	0.64
MEL_001-2b	1	2	b	2.68
MEL_002-1	2	1	a	0.78
MEL_002-3	2	3	a	2.35
MEL_002-5-A	2	5	a	1.88
MEL_002-7-B	2	7	a	1.30
MEL_002-8	2	8	a	0.65
MEL_003-1c	3	1	c	1.69
MEL_004-1	4	1	a	0.52
MEL_004-2	4	2	a	1.11
MEL_004-3	4	3	a	2.85
MEL_004-5	4	5	a	1.33
MEL_005-3	5	3	a	0.55
MEL_005-5	5	5	a	3.10
MEL_005-7	5	7	a	3.49
MEL_005-8-A	5	8	a	1.25
MEL_006-1	6	1	a	0.80
MEL_006-3	6	3	a	2.82
MEL_006-6	6	6	a	0.13
MEL_006-7	6	7	a	2.12
MEL_006-8	6	8	a	1.61
MEL_007-1b	7	1	b	0.65
MEL_007-2b	7	2	b	1.42
MEL_008-2	8	2	a	0.24
MEL_008-3	8	3	a	0.43
MEL_008-4	8	4	a	0.47
MEL_009b-2	009b	2	a	0.33
MEL_010-1aA	10	1	aA	0.47
MEL_010-1b	10	1	b	1.63
MEL_010-3	10	3	a	1.63
MEL_010-5	10	5	a	0.83
MEL_011-1b	11	1	b	1.67
MEL_011-2a	11	2	a	2.02
MEL_011-3	11	3	a	1.42
MEL_012-1b	12	1	b	2.25
MEL_012-2b	12	2	b	1.96
MEL_012-3	12	3	a	1.28
MEL_012-4	12	4	a	2.02
MEL_013-1a	13	1	a	0.43
MEL_013-2c	13	2	c	1.46
MEL_014-2	14	2	a	1.06
MEL_014-4	14	4	a	0.51
MEL_014-7	14	7	a	0.44
MEL_014-8	14	8	a	0.57
MEL_015-2	15	2	a	1.78
MEL_015-3	15	3	a	0.10
MEL_015-7	15	7	a	0.75
MEL_016-1	16	1	a	1.97
MEL_016-4	16	4	a	1.52
MEL_016-6	16	6	a	2.12
MEL_016-7	16	7	a	0.71
MEL_017-4	17	4	a	2.41
MEL_017-6	17	6	a	2.81
MEL_017-7	17	7	a	2.32
MEL_018-1	18	1	a	2.07
MEL_018-2	18	2	a	0.04
MEL_019-1a	19	1	a	1.17
MEL_019-4	19	4	a	0.76
MEL_019-5	19	5	a	1.24
MEL_019-6-A	19	6	a	0.81

MEL_020-1-A	20	1	a	2.61
MEL_020-3	20	3	a	2.19
MEL_020-5	20	5	a	1.66
MEL_020-7	20	7	a	1.36
MEL_021-1	21	1	a	0.88
MEL_021-2	21	2	a	0.34
MEL_021-3	21	3	a	0.25
MEL_021-7	21	7	a	1.39
MEL_022-1c	22	1	c	2.13
MEL_022-2b	22	2	b	0.87
MEL_023-2	23	2	a	2.56
MEL_023-4	23	4	a	4.00
MEL_023-5	23	5	a	1.50
MEL_023-6	23	6	a	0.84
MEL_024-2	24	2	a	2.17
MEL_025-1a-A	25	1	a	1.96
MEL_025-1b	25	1	b	1.01
MEL_025-1c-A	25	1	c	2.29
MEL_025-1d	25	1	d	1.92
MEL_025-1e	25	1	e	0.66
MEL_025-1f	25	1	f	1.29
MEL_026-1b	26	1	b	2.57
MEL_027-1e	27	1	e	2.04
MEL_028-1c	28	1	c	2.66
MEL_029-1c	29	1	c	1.61
MEL_029-2b	29	2	b	0.03
MEL_030-1b	30	1	b	0.73
MEL_030-2a	30	2	a	1.95
MEL_031-1c	31	1	c	2.62
MEL_031-2c	31	2	c	1.95
MEL_032-1	32	1	a	0.66
MEL_032-2	32	2	a	2.17
MEL_032-5	32	5	a	2.58
MEL_032-6	32	6	a	-0.34
MEL_032-8	32	8	a	1.95
MEL_033-1-A	33	1	a	1.07
MEL_033-2	33	2	a	0.51
MEL_033-4	33	4	a	2.06
MEL_033-5	33	5	a	2.03
MEL_033-7	33	7	a	2.44
MEL_034-4	34	4	a	2.14
MEL_034-5	34	5	a	0.15
MEL_034-6	34	6	a	0.65
MEL_034-7	34	7	a	0.70
MEL_035-1	35	1	a	2.46
MEL_035-2	35	2	a	0.76
MEL_035-3	35	3	a	0.81
MEL_035-7	35	7	a	2.42
MEL_035-8	35	8	a	1.95
MEL_036-1b	36	1	b	0.87
MEL_036-3	36	3	a	3.20
MEL_036-8	36	8	a	0.67
MEL_037-1a	37	1	a	2.93
MEL_037-2a	37	2	a	1.50
MEL_038-1a	38	1	a	1.86
MEL_038-2b	38	2	b	-0.12
MEL_039-1e	39	1	e	1.44
MEL_040-1c	40	1	c	2.17
MEL_040-2c	40	2	c	2.30
MEL_041-1c	41	1	c	2.41
MEL_041-2c	41	2	c	1.07
MEL_042-1b	42	1	b	1.77
MEL_043-1	43	1	a	3.21

MEL_043-2	43	2	a	1.93
MEL_043-3	43	3	a	2.18
MEL_043-5	43	5	a	1.23
MEL_043-6	43	6	a	2.65
MEL_043-7	43	7	a	1.52
MEL_044a-1	044a	1	a	1.25
MEL_044a-2	044a	2	a	1.96
MEL_044a-4	044a	4	a	3.08
MEL_044b-1b	044b	1	b	2.02
MEL_045-1b	45	1	b	0.64
MEL_046-1	46	1	a	0.78
MEL_046-2	46	2	a	1.60
MEL_046-7	46	7	a	1.39
MEL_047a-1	047a	1	a	0.38
MEL_047a-3	047a	3	a	1.37
MEL_047b-1a	047b	1	a	1.55
MEL_048-1a	48	1	a	0.97
MEL_048-1c	48	1	c	1.32
MEL_049-2	49	2	a	0.61
MEL_049-5	49	5	a	U
MEL_050-1	50	1	a	0.55
MEL_050-5	50	5	a	1.35
MEL_050-6	50	6	a	1.09
MEL_051-1d	51	1	d	1.92
MEL_052-1	52	1	a	1.22
MEL_052-3	52	3	a	0.61
MEL_052-5	52	5	a	0.56
MEL_052-7-A	52	7	a	0.92
MEL_052-8-A	52	8	a	1.62
MEL_053-1aA	53	1	aA	0.08
MEL_053-2aB	53	2	aB	2.52
MEL_053-3	53	3	a	1.03
MEL_054-1A	54	1	A	1.06
MEL_054-2	54	2	a	1.86
MEL_054-3A	54	3	A	1.32
MEL_054-7	54	7	a	0.83
MEL_054-8	54	8	a	2.09
MEL_055-1aA	55	1	aA	1.50
MEL_055-2b	55	2	b	2.03
MEL_056-1	56	1	a	2.82
MEL_056-4	56	4	a	2.03
MEL_056-5	56	5	a	0.99
MEL_056-6	56	6	a	2.40
MEL_057-1a	57	1	a	1.41
MEL_057-3	57	3	a	0.01
MEL_058-1	58	1	a	1.08
MEL_058-2	58	2	a	1.54
MEL_058-4	58	4	a	1.95
MEL_059-1a	59	1	a	0.95
MEL_059-1b	59	1	b	0.86
MEL_059-1c	59	1	c	1.52
MEL_059-1d	59	1	d	2.03
MEL_059-1e	59	1	e	1.97
MEL_059-1f	59	1	f	2.06
MEL_060-1a	60	1	a	1.37
MEL_060-1b	60	1	b	0.63
MEL_060-2bA	60	2	b	2.83
MEL_060-3	60	3	a	0.47
MEL_060-4	60	4	a	1.94
MEL_061-1	61	1	a	0.79
MEL_061-2	61	2	a	0.37
MEL_061-4A	61	4	A	0.22
MEL_061-7	61	7	a	1.45

MEL_062-1a	62	1	a	2.33
MEL_062-2	62	2	a	0.26
MEL_062-3	62	3	a	0.52
MEL_063a-2	063a	2	a	0.54
MEL_063b-1	063b	1	a	1.67
MEL_064-1a	64	1	a	1.10
MEL_064-1c	64	1	c	1.22
MEL_064-1d	64	1	d	0.10
MEL_064-1e	64	1	e	2.32
MEL_064-1f	64	1	f	0.62
MEL_065-1c	65	1	c	1.52
MEL_065-2b	65	2	b	1.78
MEL_070-1c	70	1	c	2.21
MEL_071-1a	71	1	a	2.50
MEL_071-1b	71	1	b	0.20
MEL_071-1c	71	1	c	1.04
MEL_071-1e	71	1	e	1.98
MEL_071-1f	71	1	f	0.43
MEL_072-1a	72	1	a	1.32
MEL_072-1b	72	1	b	3.04
MEL_072-1c	72	1	c	2.43
MEL_072-1d	72	1	d	2.10
MEL_072-1e	72	1	e	0.40
MEL_072-1f	72	1	f	2.60
MEL_073-3A	73	3	A	2.14
MEL_073-6	73	6	a	1.78
MEL_073-8	73	8	a	2.55
MEL_074-3	74	3	a	2.12
MEL_074-5	74	5	a	1.32
MEL_075-1b	75	1	b	1.37
MEL_075-2b	75	2	b	1.51
MEL_075-3	75	3	a	-0.01
MEL_075-4	75	4	a	2.17
MEL_076-1d	76	1	d	0.97
MEL_077-4	77	4	a	1.22
MEL_078-1cA	78	1	cA	1.38
MEL_081-1a	81	1	a	0.85
MEL_081-3	81	3	a	1.44
MEL_081-4	81	4	a	2.25
MEL_081-5	81	5	a	0.92
MEL_082-1aA	82	1	aA	1.39
MEL_082-1b	82	1	b	1.70
MEL_082-2	82	2	a	2.61
MEL_083-1	83	1	a	1.12
MEL_084-7	84	7	a	0.27
MEL_085-2b	85	2	b	1.75
MEL_085-4	85	4	a	2.32
MEL_086-3	86	3	a	1.13
MEL_086-7	86	7	a	2.46
MEL_087-1c	87	1	c	1.97
MEL_088-1	88	1	a	2.11
MEL_088-3	88	3	a	2.50
MEL_088-7	88	7	a	0.47
MEL_089-1a	89	1	a	1.48
MEL_089-2a	89	2	a	1.50
MEL_089-4	89	4	a	1.99
MEL_090-1a	90	1	a	1.98
MEL_090-2c	90	2	c	1.11
MEL_091-2	91	2	a	2.50
MEL_091-3	91	3	a	3.38
MEL_091-4	91	4	a	0.76
MEL_091-5	91	5	a	1.39
MEL_092-1	92	1	a	1.99

MEL_092-3	92	3	a	0.01
MEL_092-4	92	4	a	2.66
MEL_092-6	92	6	a	0.49
MEL_093-2	93	2	a	1.47
MEL_093-3	93	3	a	1.88
MEL_093-4	93	4	a	1.67
MEL_093-6	93	6	a	1.32
MEL_093-7	93	7	a	0.79
MEL_094-1c	94	1	c	0.79
MEL_095-1	95	1	a	1.49
MEL_095-2	95	2	a	0.37
MEL_095-6	95	6	a	2.51
MEL_095-7A	95	7	A	1.97
MEL_095-8B	95	8	B	1.78
MEL_096-1a	96	1	a	0.81
MEL_096-2	96	2	a	1.98
MEL_096-4	96	4	a	1.95
MEL_096-5	96	5	a	0.38
MEL_096-6	96	6	a	1.13
MEL_097-1	97	1	a	1.60
MEL_097-2A	97	2	A	1.81
MEL_097-3A	97	3	A	1.29
MEL_097-5	97	5	a	2.25
MEL_097-6	97	6	a	1.83
MEL_098-1	98	1	a	2.67
MEL_098-2	98	2	a	1.74
MEL_098-5B	98	5	B	0.68
MEL_099-2	99	2	a	2.19
MEL_099-5	99	5	a	1.39
MEL_099-8	99	8	a	U
MEL_100-1b	100	1	b	0.74
MEL_100-2b	100	2	b	1.58
MEL_101-1	101	1	a	1.99
MEL_102-1	102	1	a	2.31
MEL_102-3	102	3	a	0.82
MEL_102-4	102	4	a	0.51
MEL_102-5	102	5	a	3.03
MEL_103-1	103	1	a	2.31
MEL_103-2	103	2	a	2.98
MEL_103-3	103	3	a	0.39
MEL_103-8	103	8	a	3.31
MEL_104-2	104	2	a	0.38
MEL_104-3	104	3	a	2.50
MEL_104-4	104	4	a	2.77
MEL_104-7	104	7	a	0.94
MEL_105-1	105	1	a	1.65
MEL_105-3	105	3	a	0.62
MEL_105-5	105	5	a	0.67
MEL_106a-1	106a	1	a	2.00
MEL_106b-1	106b	1	a	0.99
MEL_106b-2	106b	2	a	0.33
MEL_107-1a	107	1	a	3.40
MEL_107-3a	107	3	a	2.20
MEL_108-1	108	1	a	1.94
MEL_108-2	108	2	a	1.47
MEL_108-3	108	3	a	0.69
MEL_108-4A	108	4	A	1.95
MEL_109-2	109	2	a	2.20
MEL_109-6A	109	6	A	2.09
MEL_109-7A	109	7	A	1.76
MEL_111-1	111	1	a	0.52
MEL_111-3	111	3	a	0.56
MEL_111-7	111	7	a	2.78

MEL_112-1a	112	1	a	0.76
MEL_112-4	112	4	a	0.66
MEL_112-5	112	5	a	1.88
MEL_113-1a	113	1	a	1.13
MEL_113-2	113	2	a	1.01
MEL_113-4	113	4	a	1.31
MEL_113-5	113	5	a	0.44
MEL_114-1	114	1	a	2.00
MEL_114-6	114	6	a	1.13
MEL_114-7	114	7	a	1.97
MEL_114-8	114	8	a	1.59
MEL_115-1	115	1	a	2.92
MEL_115-4	115	4	a	0.46
MEL_115-5	115	5	a	0.78
MEL_115-8	115	8	a	2.18
MEL_116-2	116	2	a	0.22
MEL_116-4A	116	4	A	1.61
MEL_116-6	116	6	a	1.99
MEL_116-7	116	7	a	1.91
MEL_117-2B	117	2	B	1.49
MEL_117-3B	117	3	B	1.04
MEL_117-5	117	5	a	1.76
MEL_118-1b	118	1	b	1.49
MEL_118-2aA	118	2	aA	1.09
MEL_118-3bA	118	3	bA	1.59
MEL_119-1	119	1	a	1.59
MEL_119-2	119	2	a	1.66
MEL_119-4	119	4	a	1.29
MEL_119-5	119	5	a	2.69
MEL_120-3	120	3	a	1.60
MEL_120-5	120	5	a	2.83
MEL_120-6	120	6	a	1.83
MEL_120-7	120	7	a	3.26
MEL_121-1b	121	1	b	0.61
MEL_121-1c	121	1	c	2.60
MEL_121-1d	121	1	d	3.09
MEL_121-1e	121	1	e	2.70
MEL_121-1f	121	1	f	1.91
MEL_122-1b	122	1	b	2.81
MEL_122-2b	122	2	b	1.96
MEL_122-4	122	4	a	1.42
MEL_123-1	123	1	a	0.95
MEL_123-2	123	2	a	2.93
MEL_123-4	123	4	a	1.75
MEL_124-2	124	2	a	1.41
MEL_124-4	124	4	a	1.12
MEL_124-5	124	5	a	0.83
MEL_124-7	124	7	a	3.46
MEL_125-1b	125	1	b	1.20
MEL_126-2	126	2	a	-0.07
MEL_126-3	126	3	a	-0.03
MEL_126-4	126	4	a	1.44
MEL_126-5	126	5	a	2.31
MEL_127-1b	127	1	b	1.90
MEL_127-2b	127	2	b	1.31
MEL_128-1	128	1	a	2.01
MEL_128-3	128	3	a	0.60
MEL_128-4	128	4	a	1.61
MEL_128-6	128	6	a	1.98
MEL_129-1	129	1	a	0.51
MEL_129-3	129	3	a	0.97
MEL_129-6	129	6	a	1.59
MEL_130-1	130	1	a	1.58

MEL_130-2	130	2	a	1.21
MEL_130-4	130	4	a	1.57
MEL_130-7A	130	7	A	0.33
MEL_131-1c	131	1	c	1.98
MEL_132-1	132	1	a	1.55
MEL_132-2	132	2	a	1.44
MEL_132-4	132	4	a	1.85
MEL_132-5	132	5	a	1.72
MEL_133-1B	133	1	B	0.91
MEL_133-2	133	2	a	2.24
MEL_133-4	133	4	a	0.86
MEL_133-7	133	7	a	0.30
MEL_134b-1a	134b	1	a	0.35
MEL_135-1c	135	1	c	0.85
MEL_136-1a	136	1	a	1.01
MEL_136-2a	136	2	a	2.06
MEL_136-3aB	136	3	aB	1.56
MEL_137a-1	137a	1	a	1.29
MEL_137b-1	137b	1	a	1.83
MEL_137b-3	137b	3	a	1.28
MEL_138-1	138	1	a	0.42
MEL_138-4	138	4	a	2.02
MEL_139-1A	139	1	A	0.54
MEL_139-2	139	2	a	2.73
MEL_139-6	139	6	a	1.69
MEL_139-7	139	7	a	1.49
MEL_140-1b	140	1	b	1.59
MEL_140-2b	140	2	b	1.65
MEL_140-3	140	3	a	0.49
MEL_141-1	141	1	a	0.45
MEL_141-4	141	4	a	1.22
MEL_141-6	141	6	a	1.82
MEL_141-7	141	7	a	0.59
MEL_142-2	142	2	a	0.65
MEL_142-3	142	3	a	2.15
MEL_142-4	142	4	a	1.24
MEL_142-8	142	8	a	0.76
MEL_143-2	143	2	a	1.70
MEL_143-4	143	4	a	1.84
MEL_143-7	143	7	a	1.98
MEL_143-8	143	8	a	0.84
MEL_144-1a	144	1	a	1.91
MEL_145-2	145	2	a	1.19
MEL_145-3	145	3	a	0.58
MEL_145-4	145	4	a	0.31
MEL_145-6	145	6	a	0.91
MEL_146-1a	146	1	a	0.89
MEL_146-1b	146	1	b	0.50
MEL_147-1	147	1	a	0.67
MEL_147-5A	147	5	A	0.65
MEL_148-1a	148	1	a	1.48
MEL_148-1b	148	1	b	1.60
MEL_148-1cB	148	1	cB	2.37
MEL_148-1d	148	1	d	1.88
MEL_148-1e	148	1	e	1.83
MEL_148-1fA	148	1	fA	2.70
MEL_149-1	149	1	a	1.52
MEL_149-3	149	3	a	0.66
MEL_150-3	150	3	a	1.35
MEL_150-5	150	5	a	1.69
MEL_150-8	150	8	a	1.85
MEL_151-3	151	3	a	1.58
MEL_151-4	151	4	a	1.13

MEL_151-5	151	5	a	0.17
MEL_152-1a	152	1	a	0.90
MEL_152-2a	152	2	a	1.99
MEL_152-3B	152	3	B	1.86
MEL_152-4	152	4	a	1.39
MEL_153-1	153	1	a	0.50
MEL_154-1a	154	1	a	3.17
MEL_154-3	154	3	a	0.31
MEL_155-1a	155	1	a	2.75
MEL_155-2aA	155	2	aA	2.07
MEL_155-3a	155	3	a	1.82
MEL_156-1A	156	1	A	2.01
MEL_156-4	156	4	a	2.06
MEL_156-5	156	5	a	2.00
MEL_157-1	157	1	a	1.85
MEL_157-3	157	3	a	1.92
MEL_157-5	157	5	a	0.11
MEL_157-6	157	6	a	2.17
MEL_158-1A	158	1	A	2.17
MEL_158-2	158	2	a	0.28
MEL_158-4	158	4	a	2.23
MEL_158-7	158	7	a	1.95
MEL_158-8	158	8	a	0.40
MEL_159-2A	159	2	A	1.86
MEL_159-4	159	4	a	2.26
MEL_159-5	159	5	a	0.42
MEL_159-8	159	8	a	0.71
MEL_160-1	160	1	a	1.41
MEL_160-4	160	4	a	0.48
MEL_160-8	160	8	a	2.00
MEL_161-1	161	1	a	1.77
MEL_161-2	161	2	a	2.94
MEL_161-5	161	5	a	1.68
MEL_161-7	161	7	a	2.72
MEL_162-2	162	2	a	2.02
MEL_162-3	162	3	a	-0.12
MEL_162-4A	162	4	A	1.79
MEL_162-6	162	6	a	2.94
MEL_163-2	163	2	a	1.21
MEL_163-3	163	3	a	0.91
MEL_163-4	163	4	a	2.57
MEL_163-7	163	7	a	0.47
MEL_164-2	164	2	a	1.76
MEL_164-4A	164	4	A	2.07
MEL_164-7	164	7	a	0.44
MEL_165-1A	165	1	A	0.96
MEL_165-2A	165	2	A	2.77
MEL_165-3	165	3	a	1.77
MEL_165-4	165	4	a	1.25
MEL_165-7	165	7	a	2.34
MEL_165-8	165	8	a	0.52
MEL_166-1a	166	1	a	0.42
MEL_166-2a	166	2	a	1.03
MEL_166-3b	166	3	b	1.30
MEL_167-7	167	7	a	1.40
MEL_168-1bA	168	1	bA	1.02
MEL_168-3	168	3	a	1.54
MEL_168-4	168	4	a	2.07
MEL_169-2aA	169	2	aA	2.07
MEL_169-2cA	169	2	cA	1.34
MEL_170-1b	170	1	b	-0.03
MEL_170-3	170	3	a	0.25
MEL_170-5A	170	5	A	2.64

MEL_170-8	170	8	a	1.69
MEL_171-2c	171	2	c	1.64
MEL_172-1	172	1	a	2.07
MEL_172-4A	172	4	A	0.85
MEL_172-5	172	5	a	0.24
MEL_172-6	172	6	a	0.60
MEL_173-2	173	2	a	0.76
MEL_173-5	173	5	a	1.83
MEL_174-1	174	1	a	2.41
MEL_174-2	174	2	a	1.26
MEL_174-3A	174	3	A	1.23
MEL_174-4	174	4	a	1.28
MEL_174-8B	174	8	B	1.17
MEL_175-2A	175	2	A	1.52
MEL_175-4B	175	4	B	0.39
MEL_175-5	175	5	a	1.66
MEL_175-6A	175	6	A	1.99
MEL_175-8	175	8	a	2.00
MEL_176-1	176	1	a	2.17
MEL_176-7	176	7	a	0.45
MEL_177a-1	177a	1	a	0.44
MEL_177b-1a	177b	1	a	2.58
MEL_178-1b	178	1	b	2.67
MEL_178-2a	178	2	a	1.14
MEL_178-3a	178	3	a	2.01
MEL_179-2	179	2	a	3.27
MEL_179-3	179	3	a	1.75
MEL_179-5	179	5	a	1.85
MEL_180-1	180	1	a	1.83
MEL_180-2	180	2	a	0.74
MEL_180-5	180	5	a	2.06
MEL_180-6	180	6	a	1.01
MEL_181-1	181	1	a	2.94
MEL_181-3	181	3	a	-0.10
MEL_181-5	181	5	a	0.28
MEL_181-7	181	7	a	2.61
MEL_182-1a	182	1	a	2.26
MEL_182-3b	182	3	b	0.48
MEL_182-4	182	4	a	2.22
MEL_183-1A	183	1	A	0.54
MEL_183-2B	183	2	B	1.58
MEL_183-7	183	7	a	1.80
MEL_184-1aB	184	1	aB	2.35
MEL_185-1	185	1	a	1.98
MEL_185-2	185	2	a	U
MEL_185-3	185	3	a	1.71
MEL_185-5	185	5	a	1.33
MEL_186-2b	186	2	b	0.92
MEL_187-1b	187	1	b	1.02
MEL_187-3b	187	3	b	2.31
MEL_188-2	188	2	a	1.91
MEL_188-7	188	7	a	2.79
MEL_188-8B	188	8	B	3.13
MEL_189-2	189	2	a	0.44
MEL_189-3	189	3	a	2.22
MEL_189-5	189	5	a	0.57
MEL_189-7	189	7	a	2.55
MEL_190-2	190	2	a	0.97
MEL_190-4	190	4	a	0.89
MEL_190-7	190	7	a	1.86
MEL_190-8	190	8	a	1.79
MEL_191-1	191	1	a	2.03
MEL_191-2	191	2	a	2.00

MEL_191-3	191	3	a	1.09
MEL_191-4	191	4	a	1.40
MEL_191-6	191	6	a	1.46
MEL_192-1	192	1	a	1.29
MEL_192-4	192	4	a	0.93
MEL_192-6	192	6	a	1.16
MEL_192-7	192	7	a	0.83
MEL_193-1	193	1	a	1.46
MEL_193-2	193	2	a	0.24
MEL_193-6A	193	6	A	1.05
MEL_193-7B	193	7	B	2.08
MEL_193-8A	193	8	A	1.63
MEL_194a-4B	194a	4	B	2.73
MEL_194b-1	194b	1	a	1.02
MEL_194b-4	194b	4	a	1.98
MEL_195-1a	195	1	a	0.59
MEL_195-1cA	195	1	cA	2.13
MEL_195-1cD	195	1	cD	0.32
MEL_195-1d	195	1	d	1.63
MEL_195-1e	195	1	e	1.01
MEL_195-1f	195	1	f	2.07
MEL_196-1B	196	1	B	0.64
MEL_196-2	196	2	a	2.68
MEL_196-3	196	3	a	0.78
MEL_196-4	196	4	a	2.35
MEL_196-5	196	5	a	1.88
MEL_197-2	197	2	a	1.30
MEL_197-3	197	3	a	0.65
MEL_197-5	197	5	a	1.69
MEL_197-6	197	6	a	0.52
MEL_197-7	197	7	a	1.11
MEL_198-2	198	2	a	2.85
MEL_198-3	198	3	a	1.33
MEL_198-8	198	8	a	0.55
MEL_199-1	199	1	a	3.10
MEL_199-2	199	2	a	3.49
MEL_199-3	199	3	a	1.25
MEL_199-4	199	4	a	0.80
MEL_199-7	199	7	a	2.82
MEL_200-1b	200	1	b	0.13
MEL_200-3	200	3	a	2.12
MEL_200-5	200	5	a	1.61
MEL_201-1A	201	1	A	0.65
MEL_201-4	201	4	a	1.42
MEL_201-5	201	5	a	0.24
MEL_201-6	201	6	a	0.43
MEL_202-2	202	2	a	0.47
MEL_202-3	202	3	a	0.33
MEL_202-4	202	4	a	0.47
MEL_202-6	202	6	a	1.63
MEL_203-3	203	3	a	1.63
MEL_203-5	203	5	a	0.83
MEL_203-6	203	6	a	1.67
MEL_203-8	203	8	a	2.02
MEL_204-2	204	2	a	1.42
MEL_204-7A	204	7	A	2.25
MEL_205-1d	205	1	d	1.96
MEL_205-1eB	205	1	eB	1.28
MEL_205-1f	205	1	f	2.02
MEL_206a-1a	206a	1	a	0.43
MEL_206b-1	206b	1	a	1.46
MEL_206b-2	206b	2	a	1.06
MEL_207-3	207	3	a	0.51

MEL_207-6	207	6	a	0.44
MEL_207-7	207	7	a	0.57
MEL_208-1	208	1	a	1.78
MEL_208-2	208	2	a	0.10
MEL_208-3	208	3	a	0.75
MEL_208-4	208	4	a	1.97
MEL_208-6	208	6	a	1.52
MEL_209-1	209	1	a	2.12
MEL_209-2	209	2	a	0.71
MEL_209-4A	209	4	A	2.41
MEL_209-5B	209	5	B	2.81
MEL_209-6	209	6	a	2.32
MEL_210-1b	210	1	b	2.07
MEL_210-2a	210	2	a	0.04
MEL_210-4	210	4	a	1.17
Alchemy	NA	NA	NA	0.38
Brompton	NA	NA	NA	1.33
Claire	NA	NA	NA	0.55
Hereward	NA	NA	NA	2.17
Rialto	NA	NA	NA	0.03
Robigus	NA	NA	NA	1.07
Soissons	NA	NA	NA	2.36
Xi19	NA	NA	NA	2

Table S2 Genetic markers used for genetic mapping. Chromosome and map position are indicated. NA = not applicable.

Available for download as an Excel file at www.g3journal.org/lookup/suppl/doi:10.1534/g3.115.021584/-/DC1

Table S3 Predicted rice genes within the physical region colinear with the wheat *Snn1* locus. EP = expressed protein, HP = hypothetical protein, TE = transposable element. Rice genes in bold were identified as possessing wheat orthologues in the *Snn1* region, based either on orthology to a wheat SNP, or orthology to a gene on the same wheat GSS contig as the wheat SNP (see Table 1). The rice orthologues of the two peak *Snn1* marker are underlined.

Rice gene model	Annotation
LOC_Os05g00988	HP
LOC_Os05g00990	TE
LOC_Os05g00992	EP
LOC_Os05g00994	EP
LOC_Os05g00996	TE
LOC_Os05g00998	TE
LOC_Os05g01002	TE
LOC_Os05g01006	TE
LOC_Os05g01010	EP
**LOC_Os05g01020**	**Transcriptional repressor, putative, expressed**
**LOC_Os05g01030**	**phospholipid transporting ATP-ase**
**LOC_Os05g01040**	**serine/threonine protein kinase**
LOC_Os05g01050	DNA binding protein
**LOC_Os05g01060**	**EP**
LOC_Os05g01070	EP
**LOC_Os05g01080**	**EP**
**LOC_Os05g01090**	**PEX14, putative**
LOC_Os05g01100	EP
**LOC_Os05g01110**	**Ribosomal protein LR28**
LOC_Os05g01120	Cytochrome P450
LOC_Os05g01130	TE
LOC_Os05g01140	Methyltransferase
LOC_Os05g01150	TE
LOC_Os05g01160	TE
LOC_Os05g01170	TE
LOC_Os05g01180	Zinc knuckle family protein
LOC_Os05g01190	TE
LOC_Os05g01200	Exonuclease, putative
LOC_Os05g01210	EP
**LOC_Os05g01230**	**Zinc finger protein**
**LOC_Os05g01240**	**AML1, putative**
**LOC_Os05g01250**	**SNF7 domain protein**
LOC_Os05g01256	Helix-loop-helix DNA-binding protein, putative
LOC_Os05g01262	Translocon-associated protein
LOC_Os05g01270	Peptidyl-prolyl cis-trans isomerase, putative
**LOC_Os05g01280**	**EP**
LOC_Os05g01290	EP
LOC_Os05g01300	EP
LOC_Os05g01320	EP
LOC_Os05g01330	EP
LOC_Os05g01350	TE
LOC_Os05g01360	EP
LOC_Os05g01370	Polygalacturonase inhibitor precursor, putative
LOC_Os05g01380	Polygalacturonase inhibitor precursor, putative
LOC_Os05g01390	TE
LOC_Os05g01400	TE
LOC_Os05g01410	TE
LOC_Os05g01420	TE
LOC_Os05g01430	Polygalacturonase inhibitor precursor, putative
LOC_Os05g01440	Phosphoribosylformylglycinamidine synthase, putative
LOC_Os05g01444	Polygalacturonase inhibitor precursor, putative
LOC_Os05g01450	Eukaryotic translation initiation factor 3 subunit F, putative
LOC_Os05g01460	STIP1 homology and U box-containing protein 1, putative

LOC_Os05g01470	Methionine S-methyltransferase, putative
LOC_Os05g01480	Ras-related protein, putative
LOC_Os05g01490	Ras-related protein, putative
LOC_Os05g01500	Tubulin-specific chaperone E, putative
LOC_Os05g01510	Cytoplasmic membrane protein, putative
LOC_Os05g01520	ECT protein, putative
LOC_Os05g01530	EP
LOC_Os05g01540	Serine/arginine repetitive matrix protein 1, putative
**LOC_Os05g01550**	**ZOS5-01 - C2H2 zinc finger protein, expressed**
LOC_Os05g01560	Vacuolar ATP synthase, putative
LOC_Os05g01570	Auxin-induced protein 5NG4, putative
LOC_Os05g01580	Integral membrane protein DUF6 containing protein, expressed
LOC_Os05g01590	Heat shock protein DnaJ, putative, expressed
LOC_Os05g01600	Actin, putative, expressed
LOC_Os05g01610	FYVE zinc finger domain containing protein, expressed
LOC_Os05g01620	OsFBX155 - F-box domain containing protein, expressed
LOC_Os05g01630	OsFBX156 - F-box domain containing protein, expressed
LOC_Os05g01635	EP
LOC_Os05g01640	TE
LOC_Os05g01650	TE
LOC_Os05g01660	TE
LOC_Os05g01670	TE
LOC_Os05g01675	Photosystem I P700 chlorophyll a apoprotein A1, putative, expressed
LOC_Os05g01680	RCLEA7 - Root cap and Late embryogenesis family protein precursor
LOC_Os05g01690	EP
LOC_Os05g01700	ABC transporter, ATP-binding protein, putative, expressed
**LOC_Os05g01710**	**Transcription initiation factor IIA gamma chain, expressed**
LOC_Os05g01730	Drought induced 19 protein, putative, expressed
LOC_Os05g01750	TruB family pseudouridylate synthase, putative, expressed
LOC_Os05g01760	Lysine ketoglutarate reductase trans-splicing related 1, putative
LOC_Os05g01770	TE
**LOC_Os05g01780**	**STE kinase**
**LOC_Os05g01790**	**EP**
LOC_Os05g01810	Xylem cysteine proteinase 2 precursor, putative, expressed
LOC_Os05g01820	Cytochrome b5-like Heme/Steroid binding domain containing protein
LOC_Os05g01830	EP
LOC_Os05g01840	HP
LOC_Os05g01850	TE
LOC_Os05g01860	EP
LOC_Os05g01870	HP
LOC_Os05g01880	EP
LOC_Os05g01890	EP
LOC_Os05g01900	EP
LOC_Os05g01910	Pumilio-family RNA binding protein, putative, expressed
LOC_Os05g01920	Inhibitor I family protein, putative, expressed
LOC_Os05g01940	Zinc finger, RING-type, putative, expressed
LOC_Os05g01950	EP
LOC_Os05g01960	EP
LOC_Os05g01970	NAD dependent epimerase/dehydratase family protein, putative
LOC_Os05g01990	DEAD-box ATP-dependent RNA helicase, putative, expressed
LOC_Os05g01994	Rab5-interacting protein like, putative, expressed
**LOC_Os05g02010**	**EP**

Table S4 Wheat lines used to validate the KASP marker for SNP Excalibur_c21898_1423. KASP genotype scores are indicated: homozygous A nucleotide (A:A), homozygous G nucleotide (G:G), heterozygous (A:G). U = missing data. Note, the KASP marker assays polymorphism on the opposite strand relative to the marker on the iSelect 90k array.

	Wheat Accession	KASP genotype
1	STORM	A:A
2	FIELDER	A:A
3	JACADI	A:A
4	POSIT	A:A
5	SAREK	A:A
6	RENOWN	A:A
7	VERSAILLES	A:A
8	TALON	A:A
9	JENA	A:A
10	FENDA	G:G
11	RIEBESEL	A:A
12	EPOCH	A:A
13	GLASGOW	A:A
14	WINDSOR	A:A
15	HIGHBURY	A:A
16	PANORAMA	G:G
17	BATSMAN	A:A
18	BRIGAND	A:A
19	MADRIGAL	A:A
20	EXSEPT	A:A
21	ORESTIS	A:A
22	GOLDLACE	A:A
23	PIRANHA	A:A
24	SHIRAZ	A:A
25	BUZZER	G:G
26	LANGDALE	A:A
27	TUXEDO	A:A
28	RITMO	A:A
29	COCOON	A:A
30	KRANICH	A:A
31	COMET	A:A
32	CYBER	A:A
33	TIMARU	A:A
34	LIMERICK	G:G
35	DW930861-509	U
36	TELLUS	A:A
37	DEAN	A:A
38	AXONA	G:G
39	OBELISK	A:A
40	CONTENDER	A:A
41	A13-98	A:A
42	JERICO	A:A
43	CAPRIMUS	A:A
44	MARSHAL	A:A
45	CONVOY	G:G
46	MAVERICK	A:A
47	TRAVIX	A:A
48	ECLIPSE	A:A
49	ABELE	A:A
50	WICKHAM	A:A
51	EXPLOSIV	A:A
52	SANCERRE	A:A
53	PROPHET	A:A
54	RUBENS	A:A
55	STIGG	A:A

56	ORTON	A:A
57	KWS_PODIUM	G:G
58	AARDEN	A:A
59	EDMUNDS	A:A
60	ISIDOR	A:A
61	KWS_TARGET	A:A
62	HURLEY	A:A
63	HAYDOCK	A:A
64	CARSTENS_VIII	A:A
65	KIPLING	A:A
66	PASTICHE	A:A
67	CANADAIR	A:A
68	GALAHAD	A:A
69	FLAIR	A:A
70	VISCOUNT	A:A
71	SANDOWN	A:A
72	LONGBOW	A:A
73	NEWMARKET	A:A
74	FENMAN	A:A
75	WELLINGTON	A:A
76	PHLEBAS	G:G
77	NEXUS	A:A
78	MAXWELL	A:A
79	KWS_BOHINEN	G:G
80	HUDSON	A:A
81	DENMAN	A:A
82	CANTERBURY	A:A
83	MANDATE	A:A
84	ANVIL	A:A
85	NSL_WW13	A:A
86	TEMPLE	A:A
87	FRELON	A:A
88	BROILER	A:A
89	AVOCET	U
90	HARDI	A:A
91	LAZARUS	A:A
92	TADEPI	A:A
93	SITKA	A:A
94	OCHRE	A:A
95	HAMMER	A:A

Table S5 Parental genotypic calls for significant genetic markers that co-segregate with the peak marker, Excalibur_c21898_1423. Genotype calls with the lowest frequency in the eight founders are highlighted in grey. Kukri_c37738_417, the second most highly correlated of the twelve additional manually scored markers, is also included.

No.	SNP	Chr.	cM	$-log_{10}P$	Alchemy	Brompton	Claire	Hereward	Rialto	Robigus	Soissons	Xi19
1	Excalibur_c21898_1423	1B	8.361	55.29	2	2	2	2	2	2	0	0
2	BS00093078_51	1B	8.361	54.12	0	0	0	0	0	0	2	2
3	BS00026180_51a	1B	8.361	51.61	0	0	0	0	0	0	2	2
4	Jagger_c5878_119	1B	8.361	24.19	0	2	0	0	2	0	2	2
5	Kukri_c44369_131	1B	8.361	24.04	2	2	0	0	2	0	2	2
6	BS00071333_51	1B	8.361	24.02	0	2	0	0	2	0	2	2
7	BS00022504_51	1B	8.361	23.73	2	2	0	0	2	0	2	2
8	RAC875_c24163_155	1B	8.361	23.32	2	2	0	0	2	0	2	2
9	Excalibur_c10657_796	1B	8.361	8.70	0	0	0	0	0	0	2	0
10	BS00050522_51	1B	8.361	8.11	2	2	0	2	2	2	2	0
11	Kukri_c37738_417	1B	9.679	38.44	0	0	0	0	0	0	0	2

File S1

Supporting Text

The recent consensus map (Wang *et al.* 2014) contains some map position anomalies. Four significant SNPs appeared to be located at chromosomal positions that indicated a possible mapping error. Two of these markers were located on chromosome 1B, but ~20 cM distal to *Snn1* (Excalibur_c22958_433, 60.624 cM. BS00022180_51, 64.099 cM). The third was located on chromosome 1A (BS00067961_51, 38.111 cM), while a fourth was located on 1D (BobWhite_c4303_524). Genetic map positions for these three SNPs were investigated in the MAGIC population by identifying their most highly correlated markers using the Pearson Correlation Coefficient. Where the correlation coefficient was <0.99, D' was used to estimate map position. These analyses indicated the most likely map positions for three of these SNPs were as follows: Excalibur_c22958_433: 8.361 cM (based on a correlation of 0.99 with SNP Kukri_c44369_131). BS00022180_51: 43.858 cM (based on a correlation of 0.99 with BS00071161_51). BobWhite_c4303_524: 1B, 9.679 cM (based on correlation of 0.998 with Kukri_c37738_417). The positions of these SNPs in QTL analyses have been amended accordingly. SNP BS00067961_51 showed a correlation of 0.83 with Excalibur_c16851_835 (chr 1B, 31.040 cM). However, further analysis found it to be associated (D' = 1) with clusters of markers both on chromosome 1B (eg IAAV4194, 1B 31.094 cM) and 1A. Due to the ambiguous nature of the genetic map position of this marker, we treat it here as unmapped.
