# Supplementary material for: Fine-Mapping the Wheat Snn1 Locus Conferring Sensitivity to the Parastagonospora nodorum Necrotrophic Effector SnTox1 Using an Eight Founder Multiparent Advanced Generation Inter-Cross Population
Source: G3 (Bethesda). 2015 Sep 24;5(11):2257–66. doi: 10.1534/g3.115.021584 (PMC4632045; doi:10.1534/g3.115.021584)
Supplement: Supporting Information [file supp_g3.115.021584_FigureS1.pdf]

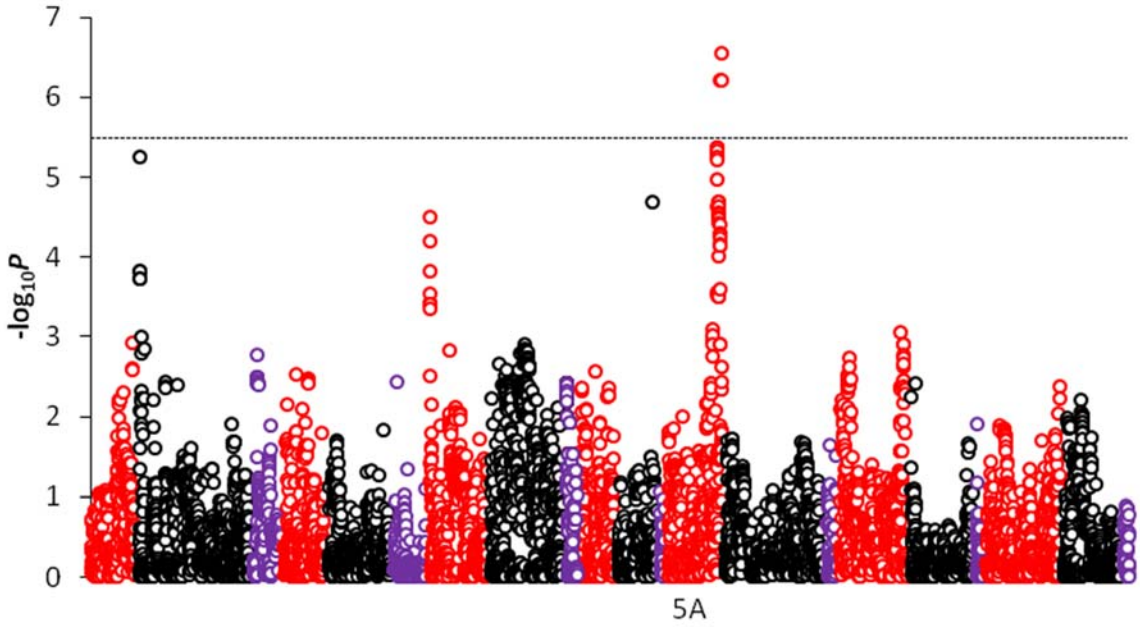

Figure S1 QTL analysis for SnTox1 sensitivity, after inclusion of the peak 1B marker 'Excalibur_c21898_1423' as a covariate. SNPs mapping to the 21 wheat chromosomes are indicated in red (A genome), black (B genome) and purple (D genome). The Bonfferoni corrected *P* = 0.05 significance threshold is indicated (dashed line). Unmapped markers are not shown.
