# Supplementary material for: Fine-Mapping the Wheat Snn1 Locus Conferring Sensitivity to the Parastagonospora nodorum Necrotrophic Effector SnTox1 Using an Eight Founder Multiparent Advanced Generation Inter-Cross Population
Source: G3 (Bethesda). 2015 Sep 24;5(11):2257–66. doi: 10.1534/g3.115.021584 (PMC4632045; doi:10.1534/g3.115.021584)
Supplement: Supporting Information [file supp_g3.115.021584_FigureS2.pdf]

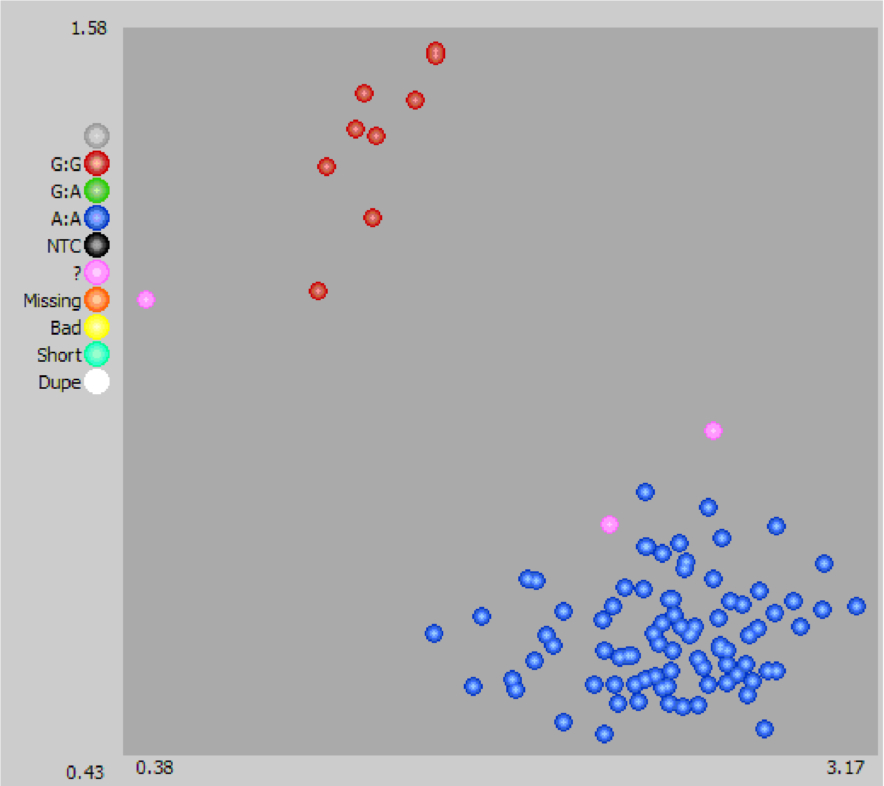

Figure S2 Conversion of SNP BS00093078_51 to the KASP genotyping platform. The assay was validated using a panel of 96 wheat varieties listed in Supplementary Table 3. Note, the KASP marker assays polymorphism on the opposite strand relative to the marker on the iSelect 90k array.
