# Supplementary material for: Fine-Mapping the Wheat Snn1 Locus Conferring Sensitivity to the Parastagonospora nodorum Necrotrophic Effector SnTox1 Using an Eight Founder Multiparent Advanced Generation Inter-Cross Population
Source: G3 (Bethesda). 2015 Sep 24;5(11):2257–66. doi: 10.1534/g3.115.021584 (PMC4632045; doi:10.1534/g3.115.021584)
Supplement: Supporting Information [file supp_g3.115.021584_TableS1.pdf]

**Table S1 MAGIC lines screened for SnTox1 sensitivity.** Adjusted phenotypic means listed.

| Variety | Funnel | Plant | Line | Mean Tox1 score |
|---|---|---|---|---|
| MEL_001-1b | 1 | 1 | b | 0.64 |
| MEL_001-2b | 1 | 2 | b | 2.68 |
| MEL_002-1 | 2 | 1 | a | 0.78 |
| MEL_002-3 | 2 | 3 | a | 2.35 |
| MEL_002-5-A | 2 | 5 | a | 1.88 |
| MEL_002-7-B | 2 | 7 | a | 1.30 |
| MEL_002-8 | 2 | 8 | a | 0.65 |
| MEL_003-1c | 3 | 1 | c | 1.69 |
| MEL_004-1 | 4 | 1 | a | 0.52 |
| MEL_004-2 | 4 | 2 | a | 1.11 |
| MEL_004-3 | 4 | 3 | a | 2.85 |
| MEL_004-5 | 4 | 5 | a | 1.33 |
| MEL_005-3 | 5 | 3 | a | 0.55 |
| MEL_005-5 | 5 | 5 | a | 3.10 |
| MEL_005-7 | 5 | 7 | a | 3.49 |
| MEL_005-8-A | 5 | 8 | a | 1.25 |
| MEL_006-1 | 6 | 1 | a | 0.80 |
| MEL_006-3 | 6 | 3 | a | 2.82 |
| MEL_006-6 | 6 | 6 | a | 0.13 |
| MEL_006-7 | 6 | 7 | a | 2.12 |
| MEL_006-8 | 6 | 8 | a | 1.61 |
| MEL_007-1b | 7 | 1 | b | 0.65 |
| MEL_007-2b | 7 | 2 | b | 1.42 |
| MEL_008-2 | 8 | 2 | a | 0.24 |
| MEL_008-3 | 8 | 3 | a | 0.43 |
| MEL_008-4 | 8 | 4 | a | 0.47 |
| MEL_009b-2 | 009b | 2 | a | 0.33 |
| MEL_010-1aA | 10 | 1 | aA | 0.47 |
| MEL_010-1b | 10 | 1 | b | 1.63 |
| MEL_010-3 | 10 | 3 | a | 1.63 |
| MEL_010-5 | 10 | 5 | a | 0.83 |
| MEL_011-1b | 11 | 1 | b | 1.67 |
| MEL_011-2a | 11 | 2 | a | 2.02 |
| MEL_011-3 | 11 | 3 | a | 1.42 |
| MEL_012-1b | 12 | 1 | b | 2.25 |
| MEL_012-2b | 12 | 2 | b | 1.96 |
| MEL_012-3 | 12 | 3 | a | 1.28 |
| MEL_012-4 | 12 | 4 | a | 2.02 |
| MEL_013-1a | 13 | 1 | a | 0.43 |
| MEL_013-2c | 13 | 2 | c | 1.46 |
| MEL_014-2 | 14 | 2 | a | 1.06 |
| MEL_014-4 | 14 | 4 | a | 0.51 |
| MEL_014-7 | 14 | 7 | a | 0.44 |
| MEL_014-8 | 14 | 8 | a | 0.57 |
| MEL_015-2 | 15 | 2 | a | 1.78 |
| MEL_015-3 | 15 | 3 | a | 0.10 |
| MEL_015-7 | 15 | 7 | a | 0.75 |
| MEL_016-1 | 16 | 1 | a | 1.97 |
| MEL_016-4 | 16 | 4 | a | 1.52 |
| MEL_016-6 | 16 | 6 | a | 2.12 |
| MEL_016-7 | 16 | 7 | a | 0.71 |
| MEL_017-4 | 17 | 4 | a | 2.41 |
| MEL_017-6 | 17 | 6 | a | 2.81 |
| MEL_017-7 | 17 | 7 | a | 2.32 |
| MEL_018-1 | 18 | 1 | a | 2.07 |
| MEL_018-2 | 18 | 2 | a | 0.04 |
| MEL_019-1a | 19 | 1 | a | 1.17 |
| MEL_019-4 | 19 | 4 | a | 0.76 |
| MEL_019-5 | 19 | 5 | a | 1.24 |
| MEL_019-6-A | 19 | 6 | a | 0.81 |

| | | | | |
|---|---|---|---|---|
| MEL_020-1-A | 20 | 1 | a | 2.61 |
| MEL_020-3 | 20 | 3 | a | 2.19 |
| MEL_020-5 | 20 | 5 | a | 1.66 |
| MEL_020-7 | 20 | 7 | a | 1.36 |
| MEL_021-1 | 21 | 1 | a | 0.88 |
| MEL_021-2 | 21 | 2 | a | 0.34 |
| MEL_021-3 | 21 | 3 | a | 0.25 |
| MEL_021-7 | 21 | 7 | a | 1.39 |
| MEL_022-1c | 22 | 1 | c | 2.13 |
| MEL_022-2b | 22 | 2 | b | 0.87 |
| MEL_023-2 | 23 | 2 | a | 2.56 |
| MEL_023-4 | 23 | 4 | a | 4.00 |
| MEL_023-5 | 23 | 5 | a | 1.50 |
| MEL_023-6 | 23 | 6 | a | 0.84 |
| MEL_024-2 | 24 | 2 | a | 2.17 |
| MEL_025-1a-A | 25 | 1 | a | 1.96 |
| MEL_025-1b | 25 | 1 | b | 1.01 |
| MEL_025-1c-A | 25 | 1 | c | 2.29 |
| MEL_025-1d | 25 | 1 | d | 1.92 |
| MEL_025-1e | 25 | 1 | e | 0.66 |
| MEL_025-1f | 25 | 1 | f | 1.29 |
| MEL_026-1b | 26 | 1 | b | 2.57 |
| MEL_027-1e | 27 | 1 | e | 2.04 |
| MEL_028-1c | 28 | 1 | c | 2.66 |
| MEL_029-1c | 29 | 1 | c | 1.61 |
| MEL_029-2b | 29 | 2 | b | 0.03 |
| MEL_030-1b | 30 | 1 | b | 0.73 |
| MEL_030-2a | 30 | 2 | a | 1.95 |
| MEL_031-1c | 31 | 1 | c | 2.62 |
| MEL_031-2c | 31 | 2 | c | 1.95 |
| MEL_032-1 | 32 | 1 | a | 0.66 |
| MEL_032-2 | 32 | 2 | a | 2.17 |
| MEL_032-5 | 32 | 5 | a | 2.58 |
| MEL_032-6 | 32 | 6 | a | -0.34 |
| MEL_032-8 | 32 | 8 | a | 1.95 |
| MEL_033-1-A | 33 | 1 | a | 1.07 |
| MEL_033-2 | 33 | 2 | a | 0.51 |
| MEL_033-4 | 33 | 4 | a | 2.06 |
| MEL_033-5 | 33 | 5 | a | 2.03 |
| MEL_033-7 | 33 | 7 | a | 2.44 |
| MEL_034-4 | 34 | 4 | a | 2.14 |
| MEL_034-5 | 34 | 5 | a | 0.15 |
| MEL_034-6 | 34 | 6 | a | 0.65 |
| MEL_034-7 | 34 | 7 | a | 0.70 |
| MEL_035-1 | 35 | 1 | a | 2.46 |
| MEL_035-2 | 35 | 2 | a | 0.76 |
| MEL_035-3 | 35 | 3 | a | 0.81 |
| MEL_035-7 | 35 | 7 | a | 2.42 |
| MEL_035-8 | 35 | 8 | a | 1.95 |
| MEL_036-1b | 36 | 1 | b | 0.87 |
| MEL_036-3 | 36 | 3 | a | 3.20 |
| MEL_036-8 | 36 | 8 | a | 0.67 |
| MEL_037-1a | 37 | 1 | a | 2.93 |
| MEL_037-2a | 37 | 2 | a | 1.50 |
| MEL_038-1a | 38 | 1 | a | 1.86 |
| MEL_038-2b | 38 | 2 | b | -0.12 |
| MEL_039-1e | 39 | 1 | e | 1.44 |
| MEL_040-1c | 40 | 1 | c | 2.17 |
| MEL_040-2c | 40 | 2 | c | 2.30 |
| MEL_041-1c | 41 | 1 | c | 2.41 |
| MEL_041-2c | 41 | 2 | c | 1.07 |
| MEL_042-1b | 42 | 1 | b | 1.77 |
| MEL_043-1 | 43 | 1 | a | 3.21 |

| | | | | |
|---|---|---|---|---|
| MEL_043-2 | 43 | 2 | a | 1.93 |
| MEL_043-3 | 43 | 3 | a | 2.18 |
| MEL_043-5 | 43 | 5 | a | 1.23 |
| MEL_043-6 | 43 | 6 | a | 2.65 |
| MEL_043-7 | 43 | 7 | a | 1.52 |
| MEL_044a-1 | 044a | 1 | a | 1.25 |
| MEL_044a-2 | 044a | 2 | a | 1.96 |
| MEL_044a-4 | 044a | 4 | a | 3.08 |
| MEL_044b-1b | 044b | 1 | b | 2.02 |
| MEL_045-1b | 45 | 1 | b | 0.64 |
| MEL_046-1 | 46 | 1 | a | 0.78 |
| MEL_046-2 | 46 | 2 | a | 1.60 |
| MEL_046-7 | 46 | 7 | a | 1.39 |
| MEL_047a-1 | 047a | 1 | a | 0.38 |
| MEL_047a-3 | 047a | 3 | a | 1.37 |
| MEL_047b-1a | 047b | 1 | a | 1.55 |
| MEL_048-1a | 48 | 1 | a | 0.97 |
| MEL_048-1c | 48 | 1 | c | 1.32 |
| MEL_049-2 | 49 | 2 | a | 0.61 |
| MEL_049-5 | 49 | 5 | a | U |
| MEL_050-1 | 50 | 1 | a | 0.55 |
| MEL_050-5 | 50 | 5 | a | 1.35 |
| MEL_050-6 | 50 | 6 | a | 1.09 |
| MEL_051-1d | 51 | 1 | d | 1.92 |
| MEL_052-1 | 52 | 1 | a | 1.22 |
| MEL_052-3 | 52 | 3 | a | 0.61 |
| MEL_052-5 | 52 | 5 | a | 0.56 |
| MEL_052-7-A | 52 | 7 | a | 0.92 |
| MEL_052-8-A | 52 | 8 | a | 1.62 |
| MEL_053-1aA | 53 | 1 | aA | 0.08 |
| MEL_053-2aB | 53 | 2 | aB | 2.52 |
| MEL_053-3 | 53 | 3 | a | 1.03 |
| MEL_054-1A | 54 | 1 | A | 1.06 |
| MEL_054-2 | 54 | 2 | a | 1.86 |
| MEL_054-3A | 54 | 3 | A | 1.32 |
| MEL_054-7 | 54 | 7 | a | 0.83 |
| MEL_054-8 | 54 | 8 | a | 2.09 |
| MEL_055-1aA | 55 | 1 | aA | 1.50 |
| MEL_055-2b | 55 | 2 | b | 2.03 |
| MEL_056-1 | 56 | 1 | a | 2.82 |
| MEL_056-4 | 56 | 4 | a | 2.03 |
| MEL_056-5 | 56 | 5 | a | 0.99 |
| MEL_056-6 | 56 | 6 | a | 2.40 |
| MEL_057-1a | 57 | 1 | a | 1.41 |
| MEL_057-3 | 57 | 3 | a | 0.01 |
| MEL_058-1 | 58 | 1 | a | 1.08 |
| MEL_058-2 | 58 | 2 | a | 1.54 |
| MEL_058-4 | 58 | 4 | a | 1.95 |
| MEL_059-1a | 59 | 1 | a | 0.95 |
| MEL_059-1b | 59 | 1 | b | 0.86 |
| MEL_059-1c | 59 | 1 | c | 1.52 |
| MEL_059-1d | 59 | 1 | d | 2.03 |
| MEL_059-1e | 59 | 1 | e | 1.97 |
| MEL_059-1f | 59 | 1 | f | 2.06 |
| MEL_060-1a | 60 | 1 | a | 1.37 |
| MEL_060-1b | 60 | 1 | b | 0.63 |
| MEL_060-2bA | 60 | 2 | b | 2.83 |
| MEL_060-3 | 60 | 3 | a | 0.47 |
| MEL_060-4 | 60 | 4 | a | 1.94 |
| MEL_061-1 | 61 | 1 | a | 0.79 |
| MEL_061-2 | 61 | 2 | a | 0.37 |
| MEL_061-4A | 61 | 4 | A | 0.22 |
| MEL_061-7 | 61 | 7 | a | 1.45 |

| | | | | |
|---|---|---|---|---|
| MEL_062-1a | 62 | 1 | a | 2.33 |
| MEL_062-2 | 62 | 2 | a | 0.26 |
| MEL_062-3 | 62 | 3 | a | 0.52 |
| MEL_063a-2 | 063a | 2 | a | 0.54 |
| MEL_063b-1 | 063b | 1 | a | 1.67 |
| MEL_064-1a | 64 | 1 | a | 1.10 |
| MEL_064-1c | 64 | 1 | c | 1.22 |
| MEL_064-1d | 64 | 1 | d | 0.10 |
| MEL_064-1e | 64 | 1 | e | 2.32 |
| MEL_064-1f | 64 | 1 | f | 0.62 |
| MEL_065-1c | 65 | 1 | c | 1.52 |
| MEL_065-2b | 65 | 2 | b | 1.78 |
| MEL_070-1c | 70 | 1 | c | 2.21 |
| MEL_071-1a | 71 | 1 | a | 2.50 |
| MEL_071-1b | 71 | 1 | b | 0.20 |
| MEL_071-1c | 71 | 1 | c | 1.04 |
| MEL_071-1e | 71 | 1 | e | 1.98 |
| MEL_071-1f | 71 | 1 | f | 0.43 |
| MEL_072-1a | 72 | 1 | a | 1.32 |
| MEL_072-1b | 72 | 1 | b | 3.04 |
| MEL_072-1c | 72 | 1 | c | 2.43 |
| MEL_072-1d | 72 | 1 | d | 2.10 |
| MEL_072-1e | 72 | 1 | e | 0.40 |
| MEL_072-1f | 72 | 1 | f | 2.60 |
| MEL_073-3A | 73 | 3 | A | 2.14 |
| MEL_073-6 | 73 | 6 | a | 1.78 |
| MEL_073-8 | 73 | 8 | a | 2.55 |
| MEL_074-3 | 74 | 3 | a | 2.12 |
| MEL_074-5 | 74 | 5 | a | 1.32 |
| MEL_075-1b | 75 | 1 | b | 1.37 |
| MEL_075-2b | 75 | 2 | b | 1.51 |
| MEL_075-3 | 75 | 3 | a | -0.01 |
| MEL_075-4 | 75 | 4 | a | 2.17 |
| MEL_076-1d | 76 | 1 | d | 0.97 |
| MEL_077-4 | 77 | 4 | a | 1.22 |
| MEL_078-1cA | 78 | 1 | cA | 1.38 |
| MEL_081-1a | 81 | 1 | a | 0.85 |
| MEL_081-3 | 81 | 3 | a | 1.44 |
| MEL_081-4 | 81 | 4 | a | 2.25 |
| MEL_081-5 | 81 | 5 | a | 0.92 |
| MEL_082-1aA | 82 | 1 | aA | 1.39 |
| MEL_082-1b | 82 | 1 | b | 1.70 |
| MEL_082-2 | 82 | 2 | a | 2.61 |
| MEL_083-1 | 83 | 1 | a | 1.12 |
| MEL_084-7 | 84 | 7 | a | 0.27 |
| MEL_085-2b | 85 | 2 | b | 1.75 |
| MEL_085-4 | 85 | 4 | a | 2.32 |
| MEL_086-3 | 86 | 3 | a | 1.13 |
| MEL_086-7 | 86 | 7 | a | 2.46 |
| MEL_087-1c | 87 | 1 | c | 1.97 |
| MEL_088-1 | 88 | 1 | a | 2.11 |
| MEL_088-3 | 88 | 3 | a | 2.50 |
| MEL_088-7 | 88 | 7 | a | 0.47 |
| MEL_089-1a | 89 | 1 | a | 1.48 |
| MEL_089-2a | 89 | 2 | a | 1.50 |
| MEL_089-4 | 89 | 4 | a | 1.99 |
| MEL_090-1a | 90 | 1 | a | 1.98 |
| MEL_090-2c | 90 | 2 | c | 1.11 |
| MEL_091-2 | 91 | 2 | a | 2.50 |
| MEL_091-3 | 91 | 3 | a | 3.38 |
| MEL_091-4 | 91 | 4 | a | 0.76 |
| MEL_091-5 | 91 | 5 | a | 1.39 |
| MEL_092-1 | 92 | 1 | a | 1.99 |

| | | | | |
|---|---|---|---|---|
| MEL_092-3 | 92 | 3 | a | 0.01 |
| MEL_092-4 | 92 | 4 | a | 2.66 |
| MEL_092-6 | 92 | 6 | a | 0.49 |
| MEL_093-2 | 93 | 2 | a | 1.47 |
| MEL_093-3 | 93 | 3 | a | 1.88 |
| MEL_093-4 | 93 | 4 | a | 1.67 |
| MEL_093-6 | 93 | 6 | a | 1.32 |
| MEL_093-7 | 93 | 7 | a | 0.79 |
| MEL_094-1c | 94 | 1 | c | 0.79 |
| MEL_095-1 | 95 | 1 | a | 1.49 |
| MEL_095-2 | 95 | 2 | a | 0.37 |
| MEL_095-6 | 95 | 6 | a | 2.51 |
| MEL_095-7A | 95 | 7 | A | 1.97 |
| MEL_095-8B | 95 | 8 | B | 1.78 |
| MEL_096-1a | 96 | 1 | a | 0.81 |
| MEL_096-2 | 96 | 2 | a | 1.98 |
| MEL_096-4 | 96 | 4 | a | 1.95 |
| MEL_096-5 | 96 | 5 | a | 0.38 |
| MEL_096-6 | 96 | 6 | a | 1.13 |
| MEL_097-1 | 97 | 1 | a | 1.60 |
| MEL_097-2A | 97 | 2 | A | 1.81 |
| MEL_097-3A | 97 | 3 | A | 1.29 |
| MEL_097-5 | 97 | 5 | a | 2.25 |
| MEL_097-6 | 97 | 6 | a | 1.83 |
| MEL_098-1 | 98 | 1 | a | 2.67 |
| MEL_098-2 | 98 | 2 | a | 1.74 |
| MEL_098-5B | 98 | 5 | B | 0.68 |
| MEL_099-2 | 99 | 2 | a | 2.19 |
| MEL_099-5 | 99 | 5 | a | 1.39 |
| MEL_099-8 | 99 | 8 | a | U |
| MEL_100-1b | 100 | 1 | b | 0.74 |
| MEL_100-2b | 100 | 2 | b | 1.58 |
| MEL_101-1 | 101 | 1 | a | 1.99 |
| MEL_102-1 | 102 | 1 | a | 2.31 |
| MEL_102-3 | 102 | 3 | a | 0.82 |
| MEL_102-4 | 102 | 4 | a | 0.51 |
| MEL_102-5 | 102 | 5 | a | 3.03 |
| MEL_103-1 | 103 | 1 | a | 2.31 |
| MEL_103-2 | 103 | 2 | a | 2.98 |
| MEL_103-3 | 103 | 3 | a | 0.39 |
| MEL_103-8 | 103 | 8 | a | 3.31 |
| MEL_104-2 | 104 | 2 | a | 0.38 |
| MEL_104-3 | 104 | 3 | a | 2.50 |
| MEL_104-4 | 104 | 4 | a | 2.77 |
| MEL_104-7 | 104 | 7 | a | 0.94 |
| MEL_105-1 | 105 | 1 | a | 1.65 |
| MEL_105-3 | 105 | 3 | a | 0.62 |
| MEL_105-5 | 105 | 5 | a | 0.67 |
| MEL_106a-1 | 106a | 1 | a | 2.00 |
| MEL_106b-1 | 106b | 1 | a | 0.99 |
| MEL_106b-2 | 106b | 2 | a | 0.33 |
| MEL_107-1a | 107 | 1 | a | 3.40 |
| MEL_107-3a | 107 | 3 | a | 2.20 |
| MEL_108-1 | 108 | 1 | a | 1.94 |
| MEL_108-2 | 108 | 2 | a | 1.47 |
| MEL_108-3 | 108 | 3 | a | 0.69 |
| MEL_108-4A | 108 | 4 | A | 1.95 |
| MEL_109-2 | 109 | 2 | a | 2.20 |
| MEL_109-6A | 109 | 6 | A | 2.09 |
| MEL_109-7A | 109 | 7 | A | 1.76 |
| MEL_111-1 | 111 | 1 | a | 0.52 |
| MEL_111-3 | 111 | 3 | a | 0.56 |
| MEL_111-7 | 111 | 7 | a | 2.78 |

| | | | | |
|---|---|---|---|---|
| MEL_112-1a | 112 | 1 | a | 0.76 |
| MEL_112-4 | 112 | 4 | a | 0.66 |
| MEL_112-5 | 112 | 5 | a | 1.88 |
| MEL_113-1a | 113 | 1 | a | 1.13 |
| MEL_113-2 | 113 | 2 | a | 1.01 |
| MEL_113-4 | 113 | 4 | a | 1.31 |
| MEL_113-5 | 113 | 5 | a | 0.44 |
| MEL_114-1 | 114 | 1 | a | 2.00 |
| MEL_114-6 | 114 | 6 | a | 1.13 |
| MEL_114-7 | 114 | 7 | a | 1.97 |
| MEL_114-8 | 114 | 8 | a | 1.59 |
| MEL_115-1 | 115 | 1 | a | 2.92 |
| MEL_115-4 | 115 | 4 | a | 0.46 |
| MEL_115-5 | 115 | 5 | a | 0.78 |
| MEL_115-8 | 115 | 8 | a | 2.18 |
| MEL_116-2 | 116 | 2 | a | 0.22 |
| MEL_116-4A | 116 | 4 | A | 1.61 |
| MEL_116-6 | 116 | 6 | a | 1.99 |
| MEL_116-7 | 116 | 7 | a | 1.91 |
| MEL_117-2B | 117 | 2 | B | 1.49 |
| MEL_117-3B | 117 | 3 | B | 1.04 |
| MEL_117-5 | 117 | 5 | a | 1.76 |
| MEL_118-1b | 118 | 1 | b | 1.49 |
| MEL_118-2aA | 118 | 2 | aA | 1.09 |
| MEL_118-3bA | 118 | 3 | bA | 1.59 |
| MEL_119-1 | 119 | 1 | a | 1.59 |
| MEL_119-2 | 119 | 2 | a | 1.66 |
| MEL_119-4 | 119 | 4 | a | 1.29 |
| MEL_119-5 | 119 | 5 | a | 2.69 |
| MEL_120-3 | 120 | 3 | a | 1.60 |
| MEL_120-5 | 120 | 5 | a | 2.83 |
| MEL_120-6 | 120 | 6 | a | 1.83 |
| MEL_120-7 | 120 | 7 | a | 3.26 |
| MEL_121-1b | 121 | 1 | b | 0.61 |
| MEL_121-1c | 121 | 1 | c | 2.60 |
| MEL_121-1d | 121 | 1 | d | 3.09 |
| MEL_121-1e | 121 | 1 | e | 2.70 |
| MEL_121-1f | 121 | 1 | f | 1.91 |
| MEL_122-1b | 122 | 1 | b | 2.81 |
| MEL_122-2b | 122 | 2 | b | 1.96 |
| MEL_122-4 | 122 | 4 | a | 1.42 |
| MEL_123-1 | 123 | 1 | a | 0.95 |
| MEL_123-2 | 123 | 2 | a | 2.93 |
| MEL_123-4 | 123 | 4 | a | 1.75 |
| MEL_124-2 | 124 | 2 | a | 1.41 |
| MEL_124-4 | 124 | 4 | a | 1.12 |
| MEL_124-5 | 124 | 5 | a | 0.83 |
| MEL_124-7 | 124 | 7 | a | 3.46 |
| MEL_125-1b | 125 | 1 | b | 1.20 |
| MEL_126-2 | 126 | 2 | a | -0.07 |
| MEL_126-3 | 126 | 3 | a | -0.03 |
| MEL_126-4 | 126 | 4 | a | 1.44 |
| MEL_126-5 | 126 | 5 | a | 2.31 |
| MEL_127-1b | 127 | 1 | b | 1.90 |
| MEL_127-2b | 127 | 2 | b | 1.31 |
| MEL_128-1 | 128 | 1 | a | 2.01 |
| MEL_128-3 | 128 | 3 | a | 0.60 |
| MEL_128-4 | 128 | 4 | a | 1.61 |
| MEL_128-6 | 128 | 6 | a | 1.98 |
| MEL_129-1 | 129 | 1 | a | 0.51 |
| MEL_129-3 | 129 | 3 | a | 0.97 |
| MEL_129-6 | 129 | 6 | a | 1.59 |
| MEL_130-1 | 130 | 1 | a | 1.58 |

| | | | | |
|---|---|---|---|---|
| MEL_130-2 | 130 | 2 | a | 1.21 |
| MEL_130-4 | 130 | 4 | a | 1.57 |
| MEL_130-7A | 130 | 7 | A | 0.33 |
| MEL_131-1c | 131 | 1 | c | 1.98 |
| MEL_132-1 | 132 | 1 | a | 1.55 |
| MEL_132-2 | 132 | 2 | a | 1.44 |
| MEL_132-4 | 132 | 4 | a | 1.85 |
| MEL_132-5 | 132 | 5 | a | 1.72 |
| MEL_133-1B | 133 | 1 | B | 0.91 |
| MEL_133-2 | 133 | 2 | a | 2.24 |
| MEL_133-4 | 133 | 4 | a | 0.86 |
| MEL_133-7 | 133 | 7 | a | 0.30 |
| MEL_134b-1a | 134b | 1 | a | 0.35 |
| MEL_135-1c | 135 | 1 | c | 0.85 |
| MEL_136-1a | 136 | 1 | a | 1.01 |
| MEL_136-2a | 136 | 2 | a | 2.06 |
| MEL_136-3aB | 136 | 3 | aB | 1.56 |
| MEL_137a-1 | 137a | 1 | a | 1.29 |
| MEL_137b-1 | 137b | 1 | a | 1.83 |
| MEL_137b-3 | 137b | 3 | a | 1.28 |
| MEL_138-1 | 138 | 1 | a | 0.42 |
| MEL_138-4 | 138 | 4 | a | 2.02 |
| MEL_139-1A | 139 | 1 | A | 0.54 |
| MEL_139-2 | 139 | 2 | a | 2.73 |
| MEL_139-6 | 139 | 6 | a | 1.69 |
| MEL_139-7 | 139 | 7 | a | 1.49 |
| MEL_140-1b | 140 | 1 | b | 1.59 |
| MEL_140-2b | 140 | 2 | b | 1.65 |
| MEL_140-3 | 140 | 3 | a | 0.49 |
| MEL_141-1 | 141 | 1 | a | 0.45 |
| MEL_141-4 | 141 | 4 | a | 1.22 |
| MEL_141-6 | 141 | 6 | a | 1.82 |
| MEL_141-7 | 141 | 7 | a | 0.59 |
| MEL_142-2 | 142 | 2 | a | 0.65 |
| MEL_142-3 | 142 | 3 | a | 2.15 |
| MEL_142-4 | 142 | 4 | a | 1.24 |
| MEL_142-8 | 142 | 8 | a | 0.76 |
| MEL_143-2 | 143 | 2 | a | 1.70 |
| MEL_143-4 | 143 | 4 | a | 1.84 |
| MEL_143-7 | 143 | 7 | a | 1.98 |
| MEL_143-8 | 143 | 8 | a | 0.84 |
| MEL_144-1a | 144 | 1 | a | 1.91 |
| MEL_145-2 | 145 | 2 | a | 1.19 |
| MEL_145-3 | 145 | 3 | a | 0.58 |
| MEL_145-4 | 145 | 4 | a | 0.31 |
| MEL_145-6 | 145 | 6 | a | 0.91 |
| MEL_146-1a | 146 | 1 | a | 0.89 |
| MEL_146-1b | 146 | 1 | b | 0.50 |
| MEL_147-1 | 147 | 1 | a | 0.67 |
| MEL_147-5A | 147 | 5 | A | 0.65 |
| MEL_148-1a | 148 | 1 | a | 1.48 |
| MEL_148-1b | 148 | 1 | b | 1.60 |
| MEL_148-1cB | 148 | 1 | cB | 2.37 |
| MEL_148-1d | 148 | 1 | d | 1.88 |
| MEL_148-1e | 148 | 1 | e | 1.83 |
| MEL_148-1fA | 148 | 1 | fA | 2.70 |
| MEL_149-1 | 149 | 1 | a | 1.52 |
| MEL_149-3 | 149 | 3 | a | 0.66 |
| MEL_150-3 | 150 | 3 | a | 1.35 |
| MEL_150-5 | 150 | 5 | a | 1.69 |
| MEL_150-8 | 150 | 8 | a | 1.85 |
| MEL_151-3 | 151 | 3 | a | 1.58 |
| MEL_151-4 | 151 | 4 | a | 1.13 |

| | | | | |
|---|---|---|---|---|
| MEL_151-5 | 151 | 5 | a | 0.17 |
| MEL_152-1a | 152 | 1 | a | 0.90 |
| MEL_152-2a | 152 | 2 | a | 1.99 |
| MEL_152-3B | 152 | 3 | B | 1.86 |
| MEL_152-4 | 152 | 4 | a | 1.39 |
| MEL_153-1 | 153 | 1 | a | 0.50 |
| MEL_154-1a | 154 | 1 | a | 3.17 |
| MEL_154-3 | 154 | 3 | a | 0.31 |
| MEL_155-1a | 155 | 1 | a | 2.75 |
| MEL_155-2aA | 155 | 2 | aA | 2.07 |
| MEL_155-3a | 155 | 3 | a | 1.82 |
| MEL_156-1A | 156 | 1 | A | 2.01 |
| MEL_156-4 | 156 | 4 | a | 2.06 |
| MEL_156-5 | 156 | 5 | a | 2.00 |
| MEL_157-1 | 157 | 1 | a | 1.85 |
| MEL_157-3 | 157 | 3 | a | 1.92 |
| MEL_157-5 | 157 | 5 | a | 0.11 |
| MEL_157-6 | 157 | 6 | a | 2.17 |
| MEL_158-1A | 158 | 1 | A | 2.17 |
| MEL_158-2 | 158 | 2 | a | 0.28 |
| MEL_158-4 | 158 | 4 | a | 2.23 |
| MEL_158-7 | 158 | 7 | a | 1.95 |
| MEL_158-8 | 158 | 8 | a | 0.40 |
| MEL_159-2A | 159 | 2 | A | 1.86 |
| MEL_159-4 | 159 | 4 | a | 2.26 |
| MEL_159-5 | 159 | 5 | a | 0.42 |
| MEL_159-8 | 159 | 8 | a | 0.71 |
| MEL_160-1 | 160 | 1 | a | 1.41 |
| MEL_160-4 | 160 | 4 | a | 0.48 |
| MEL_160-8 | 160 | 8 | a | 2.00 |
| MEL_161-1 | 161 | 1 | a | 1.77 |
| MEL_161-2 | 161 | 2 | a | 2.94 |
| MEL_161-5 | 161 | 5 | a | 1.68 |
| MEL_161-7 | 161 | 7 | a | 2.72 |
| MEL_162-2 | 162 | 2 | a | 2.02 |
| MEL_162-3 | 162 | 3 | a | -0.12 |
| MEL_162-4A | 162 | 4 | A | 1.79 |
| MEL_162-6 | 162 | 6 | a | 2.94 |
| MEL_163-2 | 163 | 2 | a | 1.21 |
| MEL_163-3 | 163 | 3 | a | 0.91 |
| MEL_163-4 | 163 | 4 | a | 2.57 |
| MEL_163-7 | 163 | 7 | a | 0.47 |
| MEL_164-2 | 164 | 2 | a | 1.76 |
| MEL_164-4A | 164 | 4 | A | 2.07 |
| MEL_164-7 | 164 | 7 | a | 0.44 |
| MEL_165-1A | 165 | 1 | A | 0.96 |
| MEL_165-2A | 165 | 2 | A | 2.77 |
| MEL_165-3 | 165 | 3 | a | 1.77 |
| MEL_165-4 | 165 | 4 | a | 1.25 |
| MEL_165-7 | 165 | 7 | a | 2.34 |
| MEL_165-8 | 165 | 8 | a | 0.52 |
| MEL_166-1a | 166 | 1 | a | 0.42 |
| MEL_166-2a | 166 | 2 | a | 1.03 |
| MEL_166-3b | 166 | 3 | b | 1.30 |
| MEL_167-7 | 167 | 7 | a | 1.40 |
| MEL_168-1bA | 168 | 1 | bA | 1.02 |
| MEL_168-3 | 168 | 3 | a | 1.54 |
| MEL_168-4 | 168 | 4 | a | 2.07 |
| MEL_169-2aA | 169 | 2 | aA | 2.07 |
| MEL_169-2cA | 169 | 2 | cA | 1.34 |
| MEL_170-1b | 170 | 1 | b | -0.03 |
| MEL_170-3 | 170 | 3 | a | 0.25 |
| MEL_170-5A | 170 | 5 | A | 2.64 |

| | | | | |
|---|---|---|---|---|
| MEL_170-8 | 170 | 8 | a | 1.69 |
| MEL_171-2c | 171 | 2 | c | 1.64 |
| MEL_172-1 | 172 | 1 | a | 2.07 |
| MEL_172-4A | 172 | 4 | A | 0.85 |
| MEL_172-5 | 172 | 5 | a | 0.24 |
| MEL_172-6 | 172 | 6 | a | 0.60 |
| MEL_173-2 | 173 | 2 | a | 0.76 |
| MEL_173-5 | 173 | 5 | a | 1.83 |
| MEL_174-1 | 174 | 1 | a | 2.41 |
| MEL_174-2 | 174 | 2 | a | 1.26 |
| MEL_174-3A | 174 | 3 | A | 1.23 |
| MEL_174-4 | 174 | 4 | a | 1.28 |
| MEL_174-8B | 174 | 8 | B | 1.17 |
| MEL_175-2A | 175 | 2 | A | 1.52 |
| MEL_175-4B | 175 | 4 | B | 0.39 |
| MEL_175-5 | 175 | 5 | a | 1.66 |
| MEL_175-6A | 175 | 6 | A | 1.99 |
| MEL_175-8 | 175 | 8 | a | 2.00 |
| MEL_176-1 | 176 | 1 | a | 2.17 |
| MEL_176-7 | 176 | 7 | a | 0.45 |
| MEL_177a-1 | 177a | 1 | a | 0.44 |
| MEL_177b-1a | 177b | 1 | a | 2.58 |
| MEL_178-1b | 178 | 1 | b | 2.67 |
| MEL_178-2a | 178 | 2 | a | 1.14 |
| MEL_178-3a | 178 | 3 | a | 2.01 |
| MEL_179-2 | 179 | 2 | a | 3.27 |
| MEL_179-3 | 179 | 3 | a | 1.75 |
| MEL_179-5 | 179 | 5 | a | 1.85 |
| MEL_180-1 | 180 | 1 | a | 1.83 |
| MEL_180-2 | 180 | 2 | a | 0.74 |
| MEL_180-5 | 180 | 5 | a | 2.06 |
| MEL_180-6 | 180 | 6 | a | 1.01 |
| MEL_181-1 | 181 | 1 | a | 2.94 |
| MEL_181-3 | 181 | 3 | a | -0.10 |
| MEL_181-5 | 181 | 5 | a | 0.28 |
| MEL_181-7 | 181 | 7 | a | 2.61 |
| MEL_182-1a | 182 | 1 | a | 2.26 |
| MEL_182-3b | 182 | 3 | b | 0.48 |
| MEL_182-4 | 182 | 4 | a | 2.22 |
| MEL_183-1A | 183 | 1 | A | 0.54 |
| MEL_183-2B | 183 | 2 | B | 1.58 |
| MEL_183-7 | 183 | 7 | a | 1.80 |
| MEL_184-1aB | 184 | 1 | aB | 2.35 |
| MEL_185-1 | 185 | 1 | a | 1.98 |
| MEL_185-2 | 185 | 2 | a | U |
| MEL_185-3 | 185 | 3 | a | 1.71 |
| MEL_185-5 | 185 | 5 | a | 1.33 |
| MEL_186-2b | 186 | 2 | b | 0.92 |
| MEL_187-1b | 187 | 1 | b | 1.02 |
| MEL_187-3b | 187 | 3 | b | 2.31 |
| MEL_188-2 | 188 | 2 | a | 1.91 |
| MEL_188-7 | 188 | 7 | a | 2.79 |
| MEL_188-8B | 188 | 8 | B | 3.13 |
| MEL_189-2 | 189 | 2 | a | 0.44 |
| MEL_189-3 | 189 | 3 | a | 2.22 |
| MEL_189-5 | 189 | 5 | a | 0.57 |
| MEL_189-7 | 189 | 7 | a | 2.55 |
| MEL_190-2 | 190 | 2 | a | 0.97 |
| MEL_190-4 | 190 | 4 | a | 0.89 |
| MEL_190-7 | 190 | 7 | a | 1.86 |
| MEL_190-8 | 190 | 8 | a | 1.79 |
| MEL_191-1 | 191 | 1 | a | 2.03 |
| MEL_191-2 | 191 | 2 | a | 2.00 |

| | | | | |
|---|---|---|---|---|
| MEL_191-3 | 191 | 3 | a | 1.09 |
| MEL_191-4 | 191 | 4 | a | 1.40 |
| MEL_191-6 | 191 | 6 | a | 1.46 |
| MEL_192-1 | 192 | 1 | a | 1.29 |
| MEL_192-4 | 192 | 4 | a | 0.93 |
| MEL_192-6 | 192 | 6 | a | 1.16 |
| MEL_192-7 | 192 | 7 | a | 0.83 |
| MEL_193-1 | 193 | 1 | a | 1.46 |
| MEL_193-2 | 193 | 2 | a | 0.24 |
| MEL_193-6A | 193 | 6 | A | 1.05 |
| MEL_193-7B | 193 | 7 | B | 2.08 |
| MEL_193-8A | 193 | 8 | A | 1.63 |
| MEL_194a-4B | 194a | 4 | B | 2.73 |
| MEL_194b-1 | 194b | 1 | a | 1.02 |
| MEL_194b-4 | 194b | 4 | a | 1.98 |
| MEL_195-1a | 195 | 1 | a | 0.59 |
| MEL_195-1cA | 195 | 1 | cA | 2.13 |
| MEL_195-1cD | 195 | 1 | cD | 0.32 |
| MEL_195-1d | 195 | 1 | d | 1.63 |
| MEL_195-1e | 195 | 1 | e | 1.01 |
| MEL_195-1f | 195 | 1 | f | 2.07 |
| MEL_196-1B | 196 | 1 | B | 0.64 |
| MEL_196-2 | 196 | 2 | a | 2.68 |
| MEL_196-3 | 196 | 3 | a | 0.78 |
| MEL_196-4 | 196 | 4 | a | 2.35 |
| MEL_196-5 | 196 | 5 | a | 1.88 |
| MEL_197-2 | 197 | 2 | a | 1.30 |
| MEL_197-3 | 197 | 3 | a | 0.65 |
| MEL_197-5 | 197 | 5 | a | 1.69 |
| MEL_197-6 | 197 | 6 | a | 0.52 |
| MEL_197-7 | 197 | 7 | a | 1.11 |
| MEL_198-2 | 198 | 2 | a | 2.85 |
| MEL_198-3 | 198 | 3 | a | 1.33 |
| MEL_198-8 | 198 | 8 | a | 0.55 |
| MEL_199-1 | 199 | 1 | a | 3.10 |
| MEL_199-2 | 199 | 2 | a | 3.49 |
| MEL_199-3 | 199 | 3 | a | 1.25 |
| MEL_199-4 | 199 | 4 | a | 0.80 |
| MEL_199-7 | 199 | 7 | a | 2.82 |
| MEL_200-1b | 200 | 1 | b | 0.13 |
| MEL_200-3 | 200 | 3 | a | 2.12 |
| MEL_200-5 | 200 | 5 | a | 1.61 |
| MEL_201-1A | 201 | 1 | A | 0.65 |
| MEL_201-4 | 201 | 4 | a | 1.42 |
| MEL_201-5 | 201 | 5 | a | 0.24 |
| MEL_201-6 | 201 | 6 | a | 0.43 |
| MEL_202-2 | 202 | 2 | a | 0.47 |
| MEL_202-3 | 202 | 3 | a | 0.33 |
| MEL_202-4 | 202 | 4 | a | 0.47 |
| MEL_202-6 | 202 | 6 | a | 1.63 |
| MEL_203-3 | 203 | 3 | a | 1.63 |
| MEL_203-5 | 203 | 5 | a | 0.83 |
| MEL_203-6 | 203 | 6 | a | 1.67 |
| MEL_203-8 | 203 | 8 | a | 2.02 |
| MEL_204-2 | 204 | 2 | a | 1.42 |
| MEL_204-7A | 204 | 7 | A | 2.25 |
| MEL_205-1d | 205 | 1 | d | 1.96 |
| MEL_205-1eB | 205 | 1 | eB | 1.28 |
| MEL_205-1f | 205 | 1 | f | 2.02 |
| MEL_206a-1a | 206a | 1 | a | 0.43 |
| MEL_206b-1 | 206b | 1 | a | 1.46 |
| MEL_206b-2 | 206b | 2 | a | 1.06 |
| MEL_207-3 | 207 | 3 | a | 0.51 |

| | | | | |
|---|---|---|---|---|
| MEL_207-6 | 207 | 6 | a | 0.44 |
| MEL_207-7 | 207 | 7 | a | 0.57 |
| MEL_208-1 | 208 | 1 | a | 1.78 |
| MEL_208-2 | 208 | 2 | a | 0.10 |
| MEL_208-3 | 208 | 3 | a | 0.75 |
| MEL_208-4 | 208 | 4 | a | 1.97 |
| MEL_208-6 | 208 | 6 | a | 1.52 |
| MEL_209-1 | 209 | 1 | a | 2.12 |
| MEL_209-2 | 209 | 2 | a | 0.71 |
| MEL_209-4A | 209 | 4 | A | 2.41 |
| MEL_209-5B | 209 | 5 | B | 2.81 |
| MEL_209-6 | 209 | 6 | a | 2.32 |
| MEL_210-1b | 210 | 1 | b | 2.07 |
| MEL_210-2a | 210 | 2 | a | 0.04 |
| MEL_210-4 | 210 | 4 | a | 1.17 |
| Alchemy | NA | NA | NA | 0.38 |
| Brompton | NA | NA | NA | 1.33 |
| Claire | NA | NA | NA | 0.55 |
| Hereward | NA | NA | NA | 2.17 |
| Rialto | NA | NA | NA | 0.03 |
| Robigus | NA | NA | NA | 1.07 |
| Soissons | NA | NA | NA | 2.36 |
| Xi19 | NA | NA | NA | 2 |
