# Supplementary material for: Fine-Mapping the Wheat Snn1 Locus Conferring Sensitivity to the Parastagonospora nodorum Necrotrophic Effector SnTox1 Using an Eight Founder Multiparent Advanced Generation Inter-Cross Population
Source: G3 (Bethesda). 2015 Sep 24;5(11):2257–66. doi: 10.1534/g3.115.021584 (PMC4632045; doi:10.1534/g3.115.021584)
Supplement: Supporting Information [file supp_g3.115.021584_TableS3.pdf]

Table S3 Predicted rice genes within the physical region colinear with the wheat *Snn1* locus. EP = expressed protein, HP = hypothetical protein, TE = transposable element. Rice genes in bold were identified as possessing wheat orthologues in the *Snn1* region, based either on orthology to a wheat SNP, or orthology to a gene on the same wheat GSS contig as the wheat SNP (see Table 1). The rice orthologues of the two peak *Snn1* marker are underlined.

Rice gene model	Annotation
LOC_Os05g00988	HP
LOC_Os05g00990	TE
LOC_Os05g00992	EP
LOC_Os05g00994	EP
LOC_Os05g00996	TE
LOC_Os05g00998	TE
LOC_Os05g01002	TE
LOC_Os05g01006	TE
LOC_Os05g01010	EP
**LOC_Os05g01020**	**Transcriptional repressor, putative, expressed**
**LOC_Os05g01030**	**phospholipid transporting ATP-ase**
**LOC_Os05g01040**	**serine/threonine protein kinase**
LOC_Os05g01050	DNA binding protein
**LOC_Os05g01060**	**EP**
LOC_Os05g01070	EP
**LOC_Os05g01080**	**EP**
**LOC_Os05g01090**	**PEX14, putative**
LOC_Os05g01100	EP
**LOC_Os05g01110**	**Ribosomal protein LR28**
LOC_Os05g01120	Cytochrome P450
LOC_Os05g01130	TE
LOC_Os05g01140	Methyltransferase
LOC_Os05g01150	TE
LOC_Os05g01160	TE
LOC_Os05g01170	TE
LOC_Os05g01180	Zinc knuckle family protein
LOC_Os05g01190	TE
LOC_Os05g01200	Exonuclease, putative
LOC_Os05g01210	EP
**LOC_Os05g01230**	**Zinc finger protein**
**LOC_Os05g01240**	**AML1, putative**
**LOC_Os05g01250**	**SNF7 domain protein**
LOC_Os05g01256	Helix-loop-helix DNA-binding protein, putative
LOC_Os05g01262	Translocon-associated protein
LOC_Os05g01270	Peptidyl-prolyl cis-trans isomerase, putative
**LOC_Os05g01280**	**EP**
LOC_Os05g01290	EP
LOC_Os05g01300	EP
LOC_Os05g01320	EP
LOC_Os05g01330	EP
LOC_Os05g01350	TE
LOC_Os05g01360	EP
LOC_Os05g01370	Polygalacturonase inhibitor precursor, putative
LOC_Os05g01380	Polygalacturonase inhibitor precursor, putative
LOC_Os05g01390	TE
LOC_Os05g01400	TE
LOC_Os05g01410	TE
LOC_Os05g01420	TE
LOC_Os05g01430	Polygalacturonase inhibitor precursor, putative
LOC_Os05g01440	Phosphoribosylformylglycinamidine synthase, putative
LOC_Os05g01444	Polygalacturonase inhibitor precursor, putative
LOC_Os05g01450	Eukaryotic translation initiation factor 3 subunit F, putative
LOC_Os05g01460	STIP1 homology and U box-containing protein 1, putative

LOC_Os05g01470	Methionine S-methyltransferase, putative
LOC_Os05g01480	Ras-related protein, putative
LOC_Os05g01490	Ras-related protein, putative
LOC_Os05g01500	Tubulin-specific chaperone E, putative
LOC_Os05g01510	Cytoplasmic membrane protein, putative
LOC_Os05g01520	ECT protein, putative
LOC_Os05g01530	EP
LOC_Os05g01540	Serine/arginine repetitive matrix protein 1, putative
**LOC_Os05g01550**	**ZOS5-01 - C2H2 zinc finger protein, expressed**
LOC_Os05g01560	Vacuolar ATP synthase, putative
LOC_Os05g01570	Auxin-induced protein 5NG4, putative
LOC_Os05g01580	Integral membrane protein DUF6 containing protein, expressed
LOC_Os05g01590	Heat shock protein DnaJ, putative, expressed
LOC_Os05g01600	Actin, putative, expressed
LOC_Os05g01610	FYVE zinc finger domain containing protein, expressed
LOC_Os05g01620	OsFBX155 - F-box domain containing protein, expressed
LOC_Os05g01630	OsFBX156 - F-box domain containing protein, expressed
LOC_Os05g01635	EP
LOC_Os05g01640	TE
LOC_Os05g01650	TE
LOC_Os05g01660	TE
LOC_Os05g01670	TE
LOC_Os05g01675	Photosystem I P700 chlorophyll a apoprotein A1, putative, expressed
LOC_Os05g01680	RCLEA7 - Root cap and Late embryogenesis family protein precursor
LOC_Os05g01690	EP
LOC_Os05g01700	ABC transporter, ATP-binding protein, putative, expressed
**LOC_Os05g01710**	**Transcription initiation factor IIA gamma chain, expressed**
LOC_Os05g01730	Drought induced 19 protein, putative, expressed
LOC_Os05g01750	TruB family pseudouridylate synthase, putative, expressed
LOC_Os05g01760	Lysine ketoglutarate reductase trans-splicing related 1, putative
LOC_Os05g01770	TE
**LOC_Os05g01780**	**STE kinase**
**LOC_Os05g01790**	**EP**
LOC_Os05g01810	Xylem cysteine proteinase 2 precursor, putative, expressed
LOC_Os05g01820	Cytochrome b5-like Heme/Steroid binding domain containing protein
LOC_Os05g01830	EP
LOC_Os05g01840	HP
LOC_Os05g01850	TE
LOC_Os05g01860	EP
LOC_Os05g01870	HP
LOC_Os05g01880	EP
LOC_Os05g01890	EP
LOC_Os05g01900	EP
LOC_Os05g01910	Pumilio-family RNA binding protein, putative, expressed
LOC_Os05g01920	Inhibitor I family protein, putative, expressed
LOC_Os05g01940	Zinc finger, RING-type, putative, expressed
LOC_Os05g01950	EP
LOC_Os05g01960	EP
LOC_Os05g01970	NAD dependent epimerase/dehydratase family protein, putative
LOC_Os05g01990	DEAD-box ATP-dependent RNA helicase, putative, expressed
LOC_Os05g01994	Rab5-interacting protein like, putative, expressed
**LOC_Os05g02010**	**EP**
