# Supplementary material for: Fine-Mapping the Wheat Snn1 Locus Conferring Sensitivity to the Parastagonospora nodorum Necrotrophic Effector SnTox1 Using an Eight Founder Multiparent Advanced Generation Inter-Cross Population
Source: G3 (Bethesda). 2015 Sep 24;5(11):2257–66. doi: 10.1534/g3.115.021584 (PMC4632045; doi:10.1534/g3.115.021584)
Supplement: Supporting Information [file supp_g3.115.021584_TableS4.pdf]

Table S4 Wheat lines used to validate the KASP marker for SNP Excalibur_c21898_1423. KASP genotype scores are indicated: homozygous A nucleotide (A:A), homozygous G nucleotide (G:G), heterozygous (A:G). U = missing data. Note, the KASP marker assays polymorphism on the opposite strand relative to the marker on the iSelect 90k array.

	Wheat Accession	KASP genotype
1	STORM	A:A
2	FIELDER	A:A
3	JACADI	A:A
4	POSIT	A:A
5	SAREK	A:A
6	RENOWN	A:A
7	VERSAILLES	A:A
8	TALON	A:A
9	JENA	A:A
10	FENDA	G:G
11	RIEBESEL	A:A
12	EPOCH	A:A
13	GLASGOW	A:A
14	WINDSOR	A:A
15	HIGHBURY	A:A
16	PANORAMA	G:G
17	BATSMAN	A:A
18	BRIGAND	A:A
19	MADRIGAL	A:A
20	EXSEPT	A:A
21	ORESTIS	A:A
22	GOLDLACE	A:A
23	PIRANHA	A:A
24	SHIRAZ	A:A
25	BUZZER	G:G
26	LANGDALE	A:A
27	TUXEDO	A:A
28	RITMO	A:A
29	COCOON	A:A
30	KRANICH	A:A
31	COMET	A:A
32	CYBER	A:A
33	TIMARU	A:A
34	LIMERICK	G:G
35	DW930861-509	U
36	TELLUS	A:A
37	DEAN	A:A
38	AXONA	G:G
39	OBELISK	A:A
40	CONTENDER	A:A
41	A13-98	A:A
42	JERICO	A:A
43	CAPRIMUS	A:A
44	MARSHAL	A:A
45	CONVOY	G:G
46	MAVERICK	A:A
47	TRAVIX	A:A
48	ECLIPSE	A:A
49	ABELE	A:A
50	WICKHAM	A:A
51	EXPLOSIV	A:A
52	SANCERRE	A:A
53	PROPHET	A:A
54	RUBENS	A:A
55	STIGG	A:A

56	ORTON	A:A
57	KWS_PODIUM	G:G
58	AARDEN	A:A
59	EDMUNDS	A:A
60	ISIDOR	A:A
61	KWS_TARGET	A:A
62	HURLEY	A:A
63	HAYDOCK	A:A
64	CARSTENS_VIII	A:A
65	KIPLING	A:A
66	PASTICHE	A:A
67	CANADAIR	A:A
68	GALAHAD	A:A
69	FLAIR	A:A
70	VISCOUNT	A:A
71	SANDOWN	A:A
72	LONGBOW	A:A
73	NEWMARKET	A:A
74	FENMAN	A:A
75	WELLINGTON	A:A
76	PHLEBAS	G:G
77	NEXUS	A:A
78	MAXWELL	A:A
79	KWS_BOHINEN	G:G
80	HUDSON	A:A
81	DENMAN	A:A
82	CANTERBURY	A:A
83	MANDATE	A:A
84	ANVIL	A:A
85	NSL_WW13	A:A
86	TEMPLE	A:A
87	FRELON	A:A
88	BROILER	A:A
89	AVOCET	U
90	HARDI	A:A
91	LAZARUS	A:A
92	TADEPI	A:A
93	SITKA	A:A
94	OCHRE	A:A
95	HAMMER	A:A
