# Supplementary material for: Fine-Mapping the Wheat Snn1 Locus Conferring Sensitivity to the Parastagonospora nodorum Necrotrophic Effector SnTox1 Using an Eight Founder Multiparent Advanced Generation Inter-Cross Population
Source: G3 (Bethesda). 2015 Sep 24;5(11):2257–66. doi: 10.1534/g3.115.021584 (PMC4632045; doi:10.1534/g3.115.021584)
Supplement: Supporting Information [file supp_g3.115.021584_TableS5.pdf]

Table S5 Parental genotypic calls for significant genetic markers that co-segregate with the peak marker, Excalibur_c21898_1423. Genotype calls with the lowest frequency in the eight founders are highlighted in grey. Kukri_c37738_417, the second most highly correlated of the twelve additional manually scored markers, is also included.

No.	SNP	Chr.	cM	$-log_{10}P$	Alchemy	Brompton	Claire	Hereward	Rialto	Robigus	Soissons	Xi19
1	Excalibur_c21898_1423	1B	8.361	55.29	2	2	2	2	2	2	0	0
2	BS00093078_51	1B	8.361	54.12	0	0	0	0	0	0	2	2
3	BS00026180_51a	1B	8.361	51.61	0	0	0	0	0	0	2	2
4	Jagger_c5878_119	1B	8.361	24.19	0	2	0	0	2	0	2	2
5	Kukri_c44369_131	1B	8.361	24.04	2	2	0	0	2	0	2	2
6	BS00071333_51	1B	8.361	24.02	0	2	0	0	2	0	2	2
7	BS00022504_51	1B	8.361	23.73	2	2	0	0	2	0	2	2
8	RAC875_c24163_155	1B	8.361	23.32	2	2	0	0	2	0	2	2
9	Excalibur_c10657_796	1B	8.361	8.70	0	0	0	0	0	0	2	0
10	BS00050522_51	1B	8.361	8.11	2	2	0	2	2	2	2	0
11	Kukri_c37738_417	1B	9.679	38.44	0	0	0	0	0	0	0	2
