# Supplementary material for: Fine-Mapping the Wheat Snn1 Locus Conferring Sensitivity to the Parastagonospora nodorum Necrotrophic Effector SnTox1 Using an Eight Founder Multiparent Advanced Generation Inter-Cross Population
Source: G3 (Bethesda). 2015 Sep 24;5(11):2257–66. doi: 10.1534/g3.115.021584 (PMC4632045; doi:10.1534/g3.115.021584)
Supplement: Supporting Information [file supp_g3.115.021584_FileS1.pdf]

File S1

Supporting Text

The recent consensus map (Wang *et al.* 2014) contains some map position anomalies. Four significant SNPs appeared to be located at chromosomal positions that indicated a possible mapping error. Two of these markers were located on chromosome 1B, but ~20 cM distal to *Snn1* (Excalibur_c22958_433, 60.624 cM. BS00022180_51, 64.099 cM). The third was located on chromosome 1A (BS00067961_51, 38.111 cM), while a fourth was located on 1D (BobWhite_c4303_524). Genetic map positions for these three SNPs were investigated in the MAGIC population by identifying their most highly correlated markers using the Pearson Correlation Coefficient. Where the correlation coefficient was <0.99, D' was used to estimate map position. These analyses indicated the most likely map positions for three of these SNPs were as follows: Excalibur_c22958_433: 8.361 cM (based on a correlation of 0.99 with SNP Kukri_c44369_131). BS00022180_51: 43.858 cM (based on a correlation of 0.99 with BS00071161_51). BobWhite_c4303_524: 1B, 9.679 cM (based on correlation of 0.998 with Kukri_c37738_417). The positions of these SNPs in QTL analyses have been amended accordingly. SNP BS00067961_51 showed a correlation of 0.83 with Excalibur_c16851_835 (chr 1B, 31.040 cM). However, further analysis found it to be associated (D' = 1) with clusters of markers both on chromosome 1B (eg IAAV4194, 1B 31.094 cM) and 1A. Due to the ambiguous nature of the genetic map position of this marker, we treat it here as unmapped.
